# Single-cell atlas reveals different immune environments between stable and vulnerable atherosclerotic plaques

**DOI:** 10.3389/fimmu.2022.1085468

**Published:** 2023-01-18

**Authors:** Peicong Ge, Hao Li, Xiaolong Ya, Yiqiao Xu, Long Ma, Qiheng He, Rong Wang, Zechen Liu, Qian Zhang, Yan Zhang, Wenjing Wang, Dong Zhang, Jizong Zhao

**Affiliations:** ^1^ Department of Neurosurgery, Beijing Tiantan Hospital, Beijing, China; ^2^ China National Clinical Research Center for Neurological Diseases, Beijing, China; ^3^ Capital Medical University, Beijing, China; ^4^ Department of Biostatistics, Harvard School of Public Health, Huntington Avenue, Boston, MA, United States; ^5^ Beijing Institute of Hepatology, Beijing YouAn Hospital, Capital Medical University, Beijing, China; ^6^ Department of Neurosurgery, Beijing Hospital, Beijing, China

**Keywords:** Vulnerable plaques, single-cell, immune environments, CyTOF/mass cytometry, RNA-seq analysis

## Abstract

**Introduction:**

Regardless of the degree of stenosis, vulnerable plaque is an important cause of ischemic stroke and thrombotic complications. The changes of the immune microenvironment within plaques seem to be an important factor affecting the characteristics of the plaque. However, the differences of immune microenvironment between stable and vulnerable plaques were remained unknown.

**Methods:**

In this study, RNA-sequencing was performed on superficial temporal arteries from 5 traumatic patients and plaques from 3 atherosclerotic patients to preliminary identify the key immune response processes in plaques. Mass cytometry (CyTOF) technology was used to explore differences in immune composition between 9 vulnerable plaques and 12 stable plaques. Finally, immunofluorescence technique was used to validate our findings in the previous analysis.

**Results:**

Our results showed that more CD86+CD68+ M1 pro-inflammatory macrophages were found in vulnerable plaques, while CD4+T memory cells were mainly found in stable plaques. In addition, a CD11c+ subset of CD4+T cells with higher IFN-r secretion was found within the vulnerable plaque. In two subsets of B cells, CD19+CD20-B cells in vulnerable plaques secreted more TNF-a and IL-6, while CD19-CD20+B cells expressed more PD-1 molecules.

**Conclusion:**

In conclusion, our study suggested that M1-like macrophages are the major cell subset affecting plaque stability, while functional B cells may also contribute to plaque stability.

## Introduction

The latest Global Burden of Disease report showed that ischemic heart disease and stroke had become the leading causes of mortality. The common pathological basis was atherosclerosis, a disease of vascular stenosis, in which subendothelial resident LDL undergoes a series of oxidations to produce ox-LDL with immune effects (1–3). On the one hand, ox-LDL can be endocytosed by macrophages from the innate immune system through scavenger receptors. On the other hand, these antigens can be taken up by antigen-presenting cells. Then, it is processed and presented to lymphocytes, activating the adaptive immune response (4, 5). Multiple types of cells and the complex immune microenvironment in plaques lead to the puzzle of plaque formation and development, which are even now not fully understood.

The bifurcation of the carotid artery is the most common site of atherosclerosis. About 10% to 15% of patients with carotid artery stenosis will experience an ischemic stroke, mainly related to local thrombosis caused by plaque rupture (6–9). Usually, atherosclerotic plaques progress slowly and silently, but some plaques can rupture suddenly, leading to acute vascular events. Therefore, the concept of vulnerable plaque was introduced to identify these high-risk plaques (9). Pathological studies suggested that vulnerable plaques were often manifested as a thin fibrous cap and large eccentric necrotic core occupying approximately one-quarter of the plaque area (10, 11). The difference in the clinical process and pathological feature implies that the two types of plaques may have different changes in the immune microenvironment. In recent years, anti-inflammatory programs have successfully treated atherosclerosis (12, 13). Therefore, researchers have paid more attention to immunotherapy. Exploring the differences in immune microenvironment composition in different plaque types and finding key intervention targets will help guide the precision immunotherapy.

In this study, we used Mass Cytometry (Mass Cytometry, CyTOF), a precise, high-dimensional approach, to identify the differences in immune microenvironment between stable and vulnerable plaques. The results showed that more M1 pro-inflammatory macrophages were found in vulnerable plaques, while CD4+T memory cells were mainly found in stable plaques. In contrast, B-cell subsets and T-cells within vulnerable plaques showed higher activity. These clusters may be important for plaque vulnerability. Our study revealed the surface characteristics of these clusters in detail, which may help identify vulnerable plaque cells and understand the relevant mechanisms of rupture.

## Materials and methods

### Human specimens and ethics statements

From August 2021 to November 2021, 21 atherosclerotic patients undergoing carotid atherectomy at Beijing Tiantan Hospital were enrolled in this study after informed consent provided. The detailed clinical data of the enrolled patients are shown in **Table 1**. Plaques were obtained from each patient. According to the imaging results (CTA/MRA/CU), plaque vulnerability was determined based on imaging biomarkers (Intraplaque haemorrhage, Lipid-rich necrotic core, Neovascularisation, Carotid plaque thickness, Surface morphology and Carotid plaque volume) (14). Normal superficial temporal arteries were obtained from other 5 patients undergoing craniocerebral trauma surgery for RNA-seq analysis (See **Figure 2** for details). This research was approved by the Institutional Review Board (IRB) and Ethics Committee of Beijing Tiantan Hospital. Written informed consent was obtained from all patients.

**Table 1:** Clinical characteristics of the patients.

	Stable Plaque	Vulnerable Plaque	p-value
**Gender (male/female)**	9/3	8/1	0.810
**Age (Mean ± SD)**	64.76 ± 4.94	65.15 ± 4.76	0.492
**Hypertension**	8	6	1.000
**Diabetes**	5	2	0.640
**Hyperlipidemia**	1	0	1.000
**Smoking**	10	6	0.712
**Drinking**	8	5	0.293
**Degree of stenosis** **Severe stenosis (70~100%)** **Moderate stenosis (50~69%)**	8 4	6 3	0.681

### RNA extraction and library construction

Peripheral blood and plaque tissues from 3 patients were used for RNA-seq analysis. In addition, superficial temporal artery samples from 5 patients undergoing craniocerebral trauma surgery were used as blank controls. The plaque tissues and artery samples were washed with DPBS (Sigma-Aldrich, United States) within 1 hour after surgical resection. Then, TRIzol method was also adopted. Sequencing libraries were generated using rRNA-depleted RNA with an NEBNext Ultra Directional RNA Library Prep Kit for Illumina (NEB, MA) following the manufacturer’s recommendations. We then performed the paired-end sequencing on illumina NovaSeq 6000(illumina, USA)as recommended by the supplier. After cluster generation, the libraries were sequenced on the Illumina HiSeq platform, and 150-bp paired-end reads were generated.

### Quality control and data analysis

In order to obtain clean data, raw data in fastq format were first processed through in-house Perl scripts. Reference genome and gene model annotation files were downloaded directly from the genome website. The index of the reference genome was built using bowtie2 v2.2.8, and paired-end clean reads were aligned to the reference genome using Hisat2 v2.0.5. Hg19 RefSeq (RNA sequences, GRCh38) was downloaded from the UCSC Genome Browser (http://genome.ucsc.edu). The clean reads were aligned with both genome hg19 and transcript reference using STAR v2.2.1, and gene expression was calculated by RSEM v1.3.0 using FPKM (fragments per kilobase of exon per million fragments mapped). We then compared RNA-seq in peripheral blood between healthy individuals and atherosclerotic patients. P<0.05 indicated statistically significant difference in expression. R was used for analysis of the gene expression data.

### Plaque tissue and superficial temporal arteries single-cell dissociation

Atherosclerotic plaque tissues were washed with Dulbecco’s phosphate-buffered saline (DPBS, Sigma-Aldrich, United States) within 1h after surgery. Each specimen was digested at 37°C for 1h using miscible liquids that contain collagenase type IV (GIBCO, Gaithersburg, United States), DNase (Sigma, DN25), hyaluronidase (Sigma, H3506), collagenase type XI (Sigma, C7657) and collagenase type II (Sigma, C6885). The mixture was filtered through a 70μm cell strainers with DPBS and washed with red blood cell (RBC) lysis buffer (BD Biosciences, United States). The dissociated cell suspension was then washed once with DPBS and resuspended in 1 mL of staining buffer (DPBS containing 5% fetal bovine serum, ScienCell, United States).

### Mass cytometry

A panel of 39 antibodies designed to distinguish a broad range of immune cells was used. Antibodies were either purchased in a preconjugated form from Fluidigm (South San Francisco, United States) or purchased from Biolegend (San Diego, United States) in a purified form and conjugated inhouse using the Maxpar X8 Multimetal Labeling Kit (Fluidigm, United States) according to the manufacturer’s instructions. The antibodies and reporter isotopes are included in **Supplementary Table 1**. The samples were then washed and stained with cisplatin-195Pt (Fluidigm, 201064) as a viability dye. Cell samples were then washed and stained with cell surface antibodies for 30 min on ice. Subsequently, the samples were permeabilized at 4°C overnight and stained with intracellular antibodies for 30 min on ice. The antibody-labeled samples were washed and incubated in 0.125 nM intercalator-Ir (Fluidigm, United States) diluted in phosphate-buffered saline (PBS, Sigma-Aldrich, United States) containing 2% formaldehyde and stored at 4°C until mass cytometry examination. Before acquisition, the samples were washed with deionized water and then resuspended at a concentration of 1 x 10^6^ cells/mL in deionized water containing a 1:20 dilution of EQ Four Element Beads (Fluidigm, United States). The samples were then examined by CyTOF2 mass cytometry (Fluidigm, United States).

### CyTOF data analysis

CyTOF data were acquired in the form of.fcs files from the CyTOF2 system. The addition of EQ Four Element Beads allowed us to use the MATLAB-based normalization technique. The obtained data were uploaded to Cytobank. Firstly, beads are filtered and active cells were selected from the specific gate. Then, CD45+ cells were gated (see **Supplementary Figure 1** for details). Further analysis using the automated dimensionality reduction algorithm FlowSom by R language. The results were shown by viSNE, a visual dimensionality reduction algorithm.

### Histology and immunofluorescence staining

Plaques from 9 patients (4 vulnerable plaques and 5 stable plaques) were fixed overnight in 4% formalin (4°C) and embedded in paraffin blocks for paraffin sections. Three paraffin sections (4 um) were cut from each specimen. Hematoxylin and eosin (H&E) staining was performed. For immunofluorescence, paraffin sections were washed twice 15 min in PBS (Sigma-Aldrich, United States), permeabilized in 0.2%-0.5% Triton X-100 (Solarbio, China) and blocked in 5% normal donkey serum (Jackson Lab, United States) for 1 h and stained with primary antibody overnight. Primary antibody was detected using fluorescent-conjected second antibodies (ZSGB-BIO, China). Primary antibodies were: anti-CD68 (Abcam, United States), anti-CD86 (Abcam, United States), and anti-HLA_DR (Abcam, United States). Sections were mounted with fluorescence mounting medium (Glostrup, Denmark). Fluorescent images were acquired on a Zeiss LSM880 NLO microscope and Zeiss Axio Scope Al was used to obtain H&E images. Three fields were randomly selected from each staining and the number of fusion particles of CD68, CD86 and HLA_DR in each field was counted. The Wilcoxon rank test was used and P<0.05 indicated statistically significant difference.

## Results

### RNA-seq data from plaques and normal superficial temporal artery was analyzed to preliminarily explore the immune microenvironment in plaques

**Figure 1:**
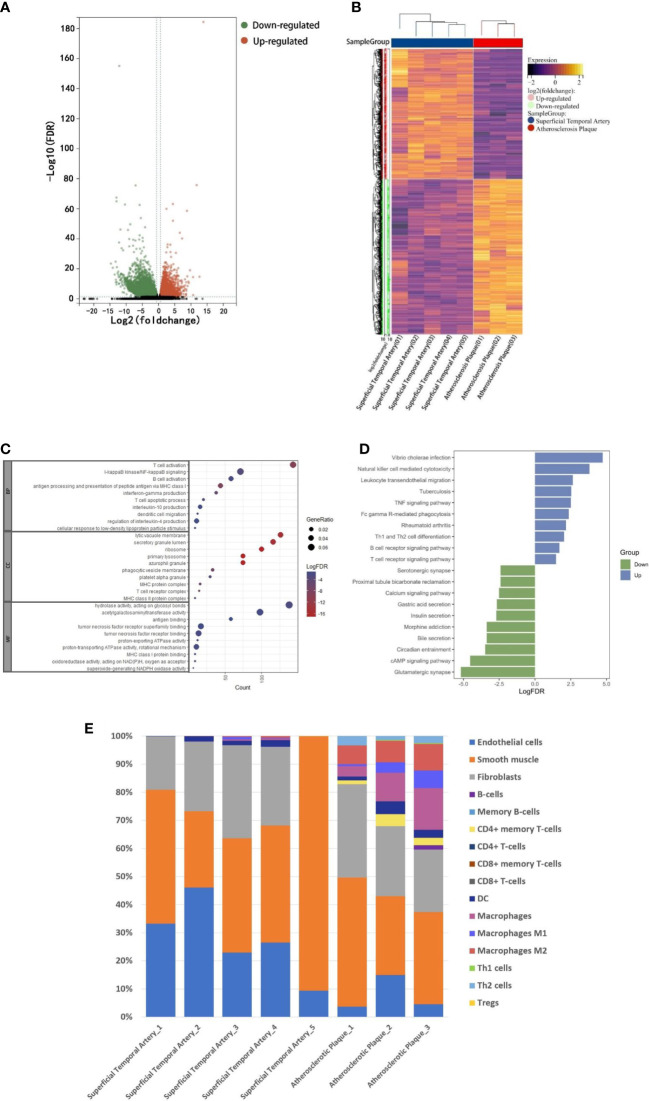
RNA-seq analysis. A: The RNA-seq data of normal superficial temporal arteries and plaques were compared. The differential genes were shown by volcanic map **(A)** and heatmap **(B)**. **(C)** Bubble map shown the GO enrichment results of up-regulated genes in plaques. **(D)** Histogram shown KEEG pathway enrichment results of up-regulated and down-regulated genes in plaques. **(E)** Immune infiltration analysis revealed the composition of superficial temporal arteries and atherosclerotic plaques.

In order to explore the composition of immune microenvironment in plaques, RNA sequencing data from plaques of 3 atherosclerotic patients and data from superficial temporal artery of 5 patients undergoing craniocerebral trauma surgery were analyzed. A total of 16036 differentially expressed genes (defined as |log2FoldChange|>1 and FDR <0.05) were identified. Among them, 3656 genes were upregulated in the atherosclerosis plaques (**Figure 1A**). To elucidate the functional implications of the differentially expressed genes, we performed pathway enrichment analyses of the upregulated and downregulated genes. For genes upregulated in plaques, NF-kappaB signaling and B cell activation were found to be significantly by Gene Ontology analysis and T cell receptor signaling pathway and B cell receptor signaling pathway were found to be significantly by Kyoto Encyclopedia of Genes and Genomes analysis. These results suggested immune responses involved T lymphocyte and B lymphocyte were existed in plaques (**Figure 1C**). Some of the up-regulated genes in plaques were also enriched in the biological processes associated with TNF signaling pathway. This suggested the TNF-a mediated immune responses within the plaques (**Figure 1D**) (Complete GO and KEEG enrichment data were shown in **Supplementary Tables 2** and **3**).

Finally, based on these RNA-Seq data, we used xCell (R package, Aran Dvir, 2017) to infer the type and proportion of cells contained in atherosclerotic plaques. The results showed that endothelial cells, smooth muscle cells and fibrous cells were composed of superficial temporal artery (**Figure 1E**). In addition to these cells, atherosclerotic plaques also contain a large number of macrophages, dendritic cells, T and B lymphocytes (**Figure 1E**). The results of immune infiltration analysis provided guidance for the subsequent selection of CyTOF antibody profiles.

### Composition of immune cells in atherosclerotic plaque

**Figure 2:**
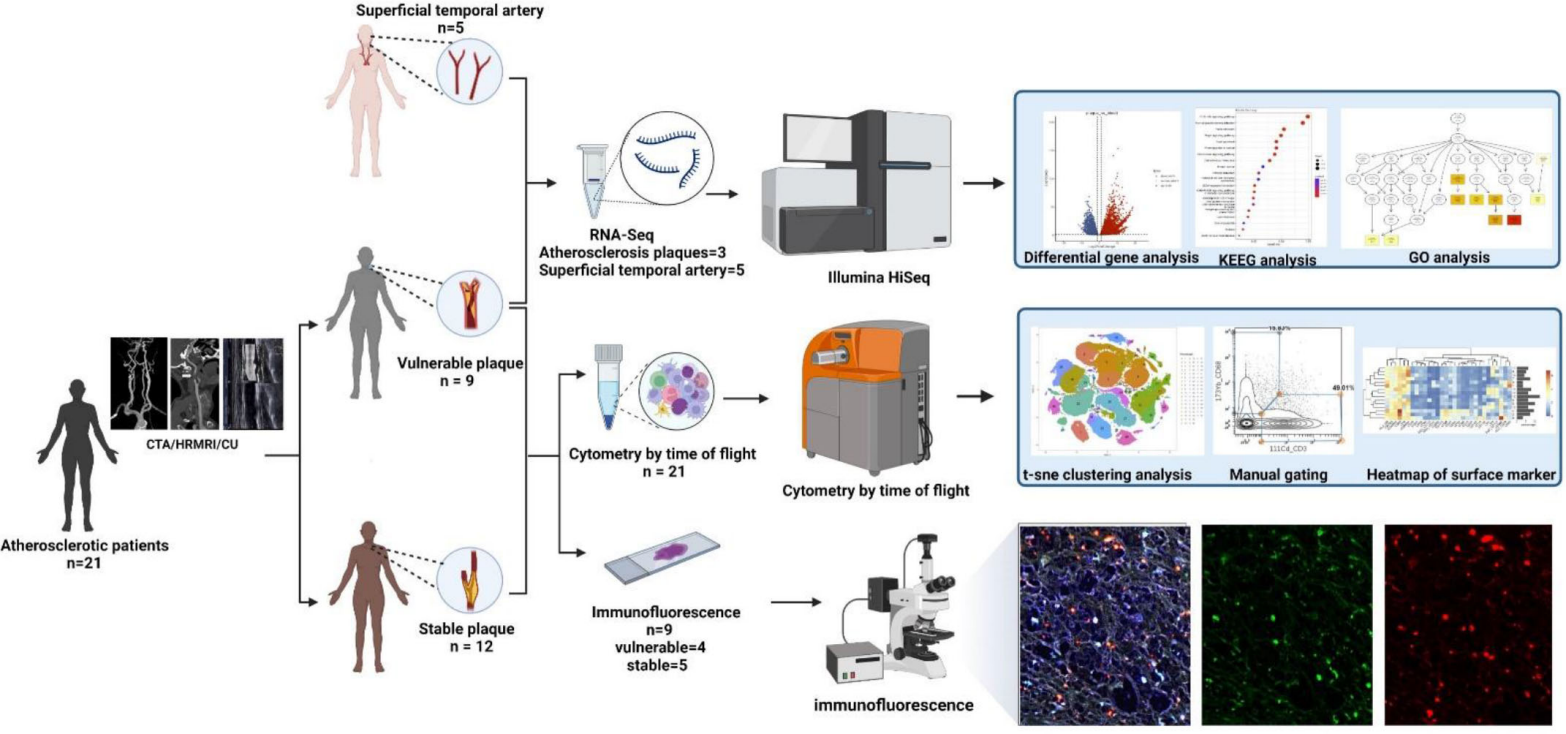
Workflow: According to preoperative imaging data, 21 enrolled atherosclerotic patients were divided into the vulnerable plaque group (n=9) and the stable plaque group (n=12). All plaques were examined by Mass Cytometry after special treatment. Among them, 9 remaining plaque samples were paraffin-embedded and were stained with immunofluorescent antibodies. Atherosclerotic plaques from 3 additional patients were performed for RNA-seq analysis. In addition, superficial temporal artery from 5 patients undergoing craniocerebral trauma surgery were also used for RNA-seq analysis as control groups (This Figure was created by Biorender).

**Figure 3:**
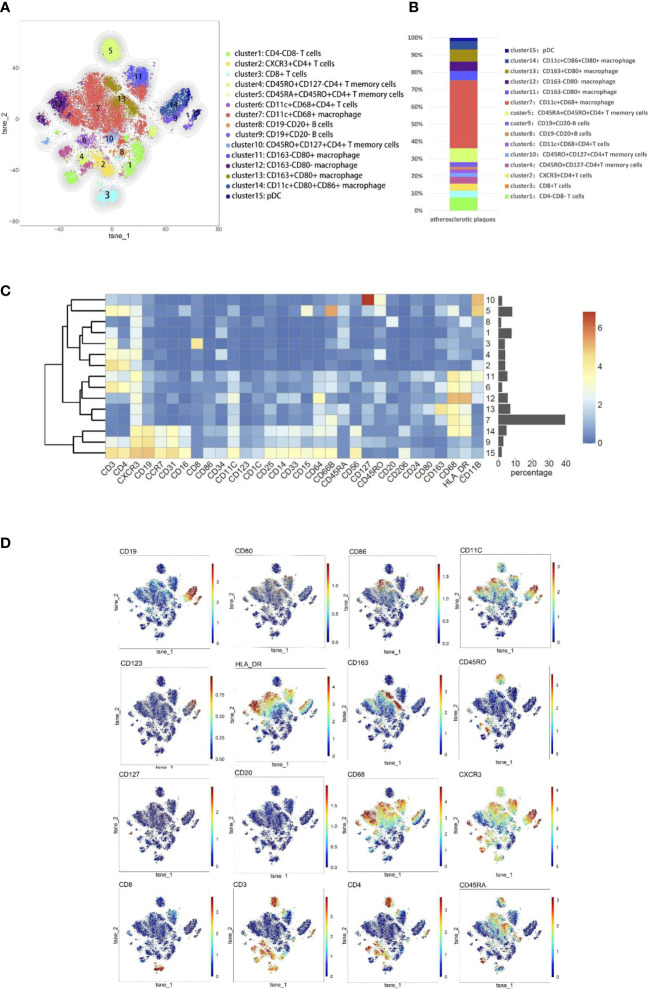
Cellular components within atherosclerotic plaques. **(A)** The analysis identified 15 populations, including T lymphocyte cells and macrophages. High-dimensional characterization of the mononuclear cell was shown by t-SNE. **(B)** The accumulation histogram presented the proportion of cell subsets within the plaques. **(C)** Heatmap showing the relative expression level of the chosen markers within the 15 cell subsets identified by the t-SNE clustering shown in **(A)**. **(D)** Markers of the particular cell were displayed by spectral colors on t-SNE maps.

According to the imaging (CTA/MRA/UTA) results, we divided the enrolled specimens into 12 stable plaques and 9 vulnerable plaques (Typical H&E images of stable and vulnerable plaques were shown in **Supplementary Figure 2**). Specific metal antibodies were selected on the basis of RNA-seq analysis (Detailed information of antibodies panel was shown in **Supplementary Table 1**). Flowsom, an unsupervised clustering method, was used to analyze the cellular composition of atherosclerotic plaques from 21 patients (**Figure 2**). Finally, 15 clusters were found. Each cell type was identified by specific markers on the its surface (**Figure 3A**). We found that macrophages and lymphocytes were the main components of plaques (**Figure 3B**). Macrophages were composed of 6 clusters, accounting for 63.9%. Lymphocytes consisted of T lymphocytes and B lymphocytes. While 7 clusters made up T lymphocytes, accounting for 31.9%. B lymphocytes were composed of two clusters, accounting for 4.2% (**Figure 3B**). T-SNE was employed to convert high-dimensional CyTOF data and was used to map the immune compartments of all samples.

### T cells were dominant in stable plaques, and macrophages were dominant in vulnerable plaques

**Figure 4:**
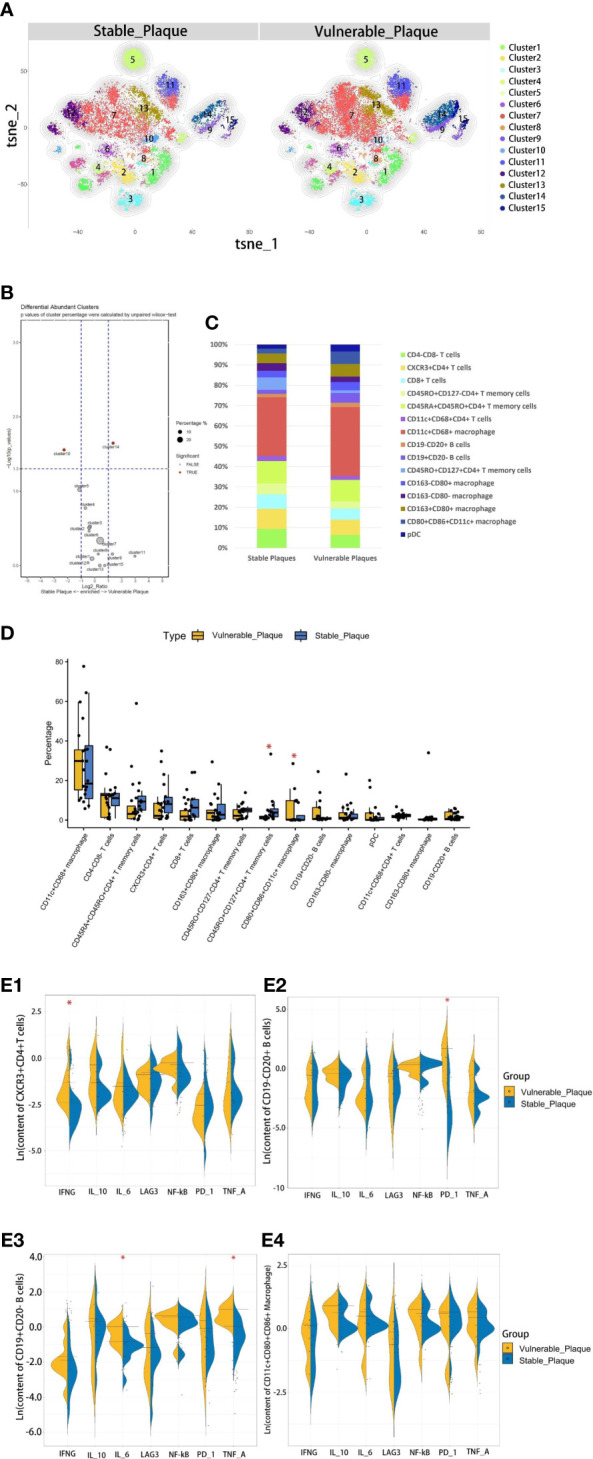
Comparison of cellular components in stable plaques and vulnerable plaques. **(A)** T-SNE plots of clusters were displayed for representative stable and vulnerable plaques. **(B)** The accumulation histogram showed the proportion of each cell subset in two types of plaques. **(C)** SPADEVizR method was used to analyze and present cell composition differences between vulnerable and stable plaques. **(D)** Bar graphs showing the changes in abundance of the cell populations identified in the t-SNE clustering, between vulnerable and stable plaques (Asterisks represented subsets of cells that were statistically different). **(E)** (1-4): The levels of functional molecules expressed by the same cell subsets within the vulnerable plaques and stable plaques (Asterisks represented subsets of cells that were statistically different).

The composition of the two plaques was compared to explore differences in immune composition between stable and vulnerable plaques. Overall, each cell subset was distributed in both types of plaques. T cells were predominant in stable plaques, accounting for 57.2%. Macrophages accounted for 39.1%. However, macrophages were the most abundant cell group in vulnerable plaques, accounting for 56.4%. T cells only accounted for 36.7% (**Figure 4C**). These seemed to suggest differences in immune environments between the two plaques.

### CD4+T memory cells were more abundant in stable plaques, while CD11c+CD4+T cells in vulnerable plaques secreted higher IFN-r

The type of T cells in plaques was diverse. CD4+T cells dominate the plaques. There were 5 clusters of CD4+T lymphocytes, including memory cells and effector cells. Comparing the percentage of cell subsets in the two plaques, CD4+T cells of cluster10 were the only group of cells more abundant in stable plaques (p<0.05) (**Figures 4B, D**). CD45RO+ and CD127, surface markers of memory cells, co-exist on the surface of cells from this cluster (15, 16). There was no difference in the content of other subsets between the two types of plaque. However, Cluster2 (CD11c+CD4+T cells) in vulnerable plaques secreted a higher level of IFN-r than in stable plaques (p< 0.05) (**Figure 4E**-1). It was suggested that this group of cells in vulnerable plaques might also play a unique function.

### CD19+CD20-B cells in vulnerable plaques secreted more TNF-a and IL-6, while CD19-CD20+B cells expressed more PD-1 molecules

Two types of B cells existed in samples. These B cells have different surface molecular patterns. Cluster9 expressed CD19+, whereas Cluster8 expressed CD20 but not CD19 (**Figure 3D**). Cluster9 also expressed surface markers HLA_DR and CCR7, which were involved in cell activation and migration, suggesting that this cluster may be a group of activated B subsets cells (17, 18). There was no difference in the number of these clusters between the two types of plaque. Functional analysis showed that cluster8 expressed a higher level of PD-1 molecule in vulnerable plaques (p<0.05) (**Figure 4E-2**). Cluster9 showed a higher level of TNF-a and IL-6 (p<0.05) (**Figure 4E-3** and **4E-4**).

### Vulnerable plaques contain more CD86+CD68+ M1 pro-inflammatory macrophages

**Figure 5:**
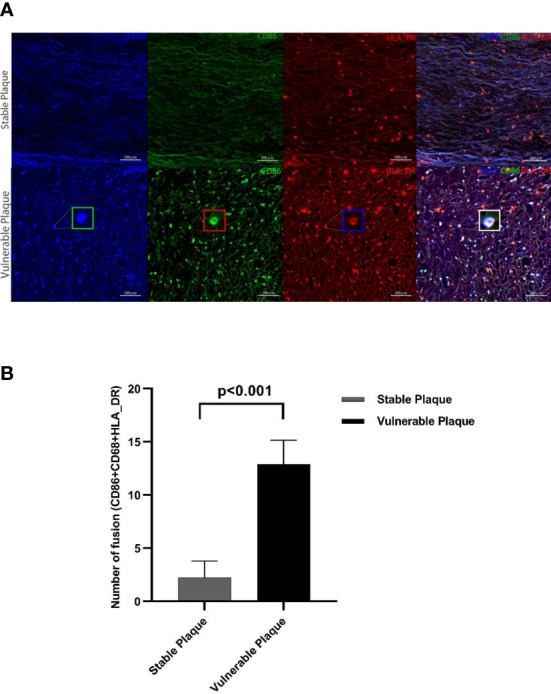
Polychromatic immunofluorescence. A larger amount of activated M1 macrophages were expressed in vulnerable plaques than in stable plaques. Typical multicolor immunofluorescence images of stable and vulnerable plaques are shown in Figure **(A)**. Statistical difference in CD68+CD80+HLA_DR+ particles between two groups were shown in Figure **(B)**.

Macrophages were the most abundant cluster in plaques and contained multiple cell subsets (**Figure 4C**). However, compared with stable plaques, cluster14 was the only cluster that was more abundant in vulnerable plaques (**Figures 4B, D**). These clusters expressed CD68 and HLA_DR, typical macrophage surface markers (19). In addition, CD86, corresponding to M1-like macrophages, existed on the surface of cluster14 (20, 21). Polychromatic immunofluorescence showed that there were more M1 macrophages in vulnerable plaques (**Figures 5A, B**). Functional analysis showed that this cluster also expressed IL-6 and IFN-r (pro-inflammatory cytokines), and functional analysis did not reveal a significant difference (**Figure 4E-4**).

## Discussion

Stroke has become a severe disease endangering human health, and its high mortality and disability rate have increased the global burden of disease (22, 23). Many of these diseases are caused by plaque shedding in the carotid artery that blocked the distal blood vessels. Deciduous plaques (often referred to as vulnerable plaques) are more likely to cause distal vascular obstruction than stable plaques (14, 24). Therefore, it is particularly important to explore the differences in immune microenvironment composition between stable plaques and vulnerable plaques. In this study, we used CyTOF technology to analyze the cell composition between two types of plaques. It was found that CD4+T memory cells were mainly present in stable plaques, while M1 macrophages were mainly existed in vulnerable plaques. Polychromatic immunofluorescence and RNA-seq analysis further confirmed this finding that was more M1 macrophages in vulnerable plaques. In subgroup analysis, different polarization directions of macrophages seemed to play an important role in the stability of plaques. In addition, CyTOF analysis also found that plaque-derived CD4+T cells and B cells showed more active function, although there was no statistical difference in their content.

As an important cell type in plaques, T lymphocytes are widely involved in the development of atherosclerotic lesions (25, 26). Comparative analysis showed that cluster2, a CD4+T cell subset, was highly secreted IFN-r in vulnerable plaques. IFN-r is the main effector molecule of Th1 cells (26–28), suggesting that cluster 2 cells in vulnerable plaques were more inclined to the pro-inflammatory Th1 activation state. IFN-r, as an important proatherogenic cytokine, plays an important role in the development of atherosclerosis (29, 30). IFN-r could promote the formation of foam cells and lead to plaque instability by affecting endothelial cell function (31–33). This implied that CD11c+ CD4+T cells with high secreted IFN-r in vulnerable plaques might be the primary cell subsets causing vulnerable transformation of plaques.

Although B cells are less abundant in plaque tissue, they are also considered important in the immune microenvironment of plaques (34, 35). We found two clusters of B lymphocytes in plaques, and there was no difference in the amount of B lymphocytes between the two types. Our study suggested that the two subsets of B cells in vulnerable plaques may play a role in plaque vulnerability in different ways. CD19+CD20- B cells from vulnerable plaques expressed the pro-inflammatory cytokines IL-6 and TNF-a. IL6 generally was consider to be a pro-atherogenic cytokine that had a strong regulatory effect on the extracellular matrix (36, 37). IL-6 stimulates the synthesis of matrix-degrading enzymes that erode the matrix within plaques, leading to the rupture of plaques (38, 39). In addition, TNF-a derived from B cells can activate macrophages within plaques to produce more TNF-a, further leading to apoptosis and causing rupture (40). This subset of B cells may influence plaque stability *via* these proinflammatory cytokines. The other CD19-CD20+ B cells from vulnerable plaques expressed higher level of PD-1. Studies have found that high expression of PD-1 by innate-type B cells after their activation by antigens and PD-1 molecule on the surface of B cells facilitate the adaptive responses through longer-lived plasma cells and memory cells generated (41). Animal experiments have also found that B2 cells involved in adaptive immune response can promote the progression of atherosclerotic plaques (42). This suggests that CD19-CD20+B cells in vulnerable plaques may mediate local adaptive immune response through PD-1 molecules on their surface and trigger plaque progression. Differences in expression levels of functional molecules expressed by the same clusters reflected the difference in immune microenvironment between stable and vulnerable plaques. These differences may be an important cause of plaque instability.

Macrophages are key players in atherosclerotic disease and their polarization states have a role in atherogenesis (43). Phagocytosis, clearance of ox-LDL, secretion of a variety of cytokines and antigen presentation were the important functions of macrophages involved in plaque lesions (44). These macrophages can change the phenotype expression, depending on the location and microenvironment. Previous studies have found that the lesions of stable plaques were mainly composed of M2-like macrophages, while M1-like macrophages primarily existed in vulnerable plaques (45). This is consistent with our findings. In this study, we found that a variety of macrophage subpopulations existed in plaques, among which cluster14 content was higher in vulnerable plaques. This subgroup expressed the surface marker of CD86 and CD68, which were surface markers of M1-like pro-inflammatory macrophages. Functional analysis showed that these cells secreted pro-inflammatory cytokines and the functional status were consistent in different type of environments. The small number of these cells in the stable plaques suggested that a weak proinflammatory response was also present in the stable plaque, The change in the number of CD86+CD68+ M1-like macrophages may disrupt the original homeostasis and promote plaque vulnerability.

This also reflected the dynamic association between vulnerable and stable plaques. Further multicolor immunofluorescence revealed the presence of more MMP2 and MMP9 within the vulnerable plaques (see **Supplementary Figure 3** for details). It is generally believed that M1 macrophages degraded the extracellular matrix by secreting proteolytic enzymes, thus causing the rupture of fibrous caps (25). This may also be a reason for the vulnerable transformation.

Compared with vulnerable plaques, the immune environment in stable plaques was relatively mild. The same subsets of cells presented in stable plaques were not as active as those in vulnerable plaques. In contrast, only CD4+T memory cells were more abundant in stable plaques. Memory cells are generally considered the evidence of a prior adaptive immune response. This evidence also suggested that atherosclerotic plaque was a chronic progressive disease. In response to reappeared antigen stimulation, memory T cells may trigger the next inflammatory response within the plaque, promoting further plaque progression.

There are also some limitations to our study. Firstly, only 32 antibodies were selected, and some subsets could not be effectively distinguished. Secondly, mononuclear cells were extracted from the specimen as a whole, which could not reflect the immune changes in different parts of the plaque. In future study, spatial single-cell technology could effectively solve this problem. Finally, atherosclerotic plaques from patients undergoing surgery may be in the terminal stage of lesion evolution, so it is necessary to analyze the immune components of plaques in different periods, and reflect the dynamic changes in plaque immune environment more comprehensively. Further and more refined exploration is needed.

## Data availability statement

The data presented in the study are deposited in the National Genomics Data Center repository, accession number OMIX002548.

## Ethics statement

The studies involving human participants were reviewed and approved by Institutional Review Board (IRB) and Ethics Committee of Beijing Tiantan Hospital. The patients/participants provided their written informed consent to participate in this study.

## Author contributions

PG conducted the experiment and wrote this article. HL designed the experiment and collected the data. XY provided the surgical specimen and analysis data. YX, QH, LM helped organize some of the data. QZ and XY performed the atherectomy. WW provided the guidance for this experiment. DZ and JZ supervised this experiment. All authors contributed to the article and approved the submitted version.
