# Supplementary material for: Single-cell atlas reveals different immune environments between stable and vulnerable atherosclerotic plaques
Source: Front Immunol. 2023 Jan 18;13:1085468. doi: 10.3389/fimmu.2022.1085468 (PMC9889979; doi:10.3389/fimmu.2022.1085468)
Supplement: Supplementary file 1 [file DataSheet_1.pdf]

Category	GOID	Descriptio	GeneRatic	BgRatio	pvalue	padj	geneID	geneNam	Count	LogFDR
BP	GO:00362	granulocy	0.075499	496/16305	8.16E-42	4.8E-38	ENSG0000	CAP1/CTS	212	-37.3186
BP	GO:00421	neutrophil	0.07443	490/16305	4.65E-41	1.37E-37	ENSG0000	CAP1/CTS	209	-36.8636
BP	GO:00024	neutrophil	0.07443	493/16305	1.43E-40	1.69E-37	ENSG0000	CAP1/CTS	209	-36.7732
BP	GO:00022	neutrophil	0.073362	481/16305	8.51E-41	1.53E-37	ENSG0000	CAP1/CTS	206	-36.8152
BP	GO:00433	neutrophil	0.073006	478/16305	1.04E-40	1.53E-37	ENSG0000	CAP1/CTS	205	-36.8152
BP	GO:00027	immune re	0.054843	495/16305	1.1E-14	4.03E-12	ENSG0000	FCGR3A/C	154	-11.3942
BP	GO:00421	T cell activ	0.050926	443/16305	3.66E-15	1.54E-12	ENSG0000	DDOST/FL	143	-11.8125
BP	GO:00026	regulation	0.049501	471/16305	1.7E-11	3.13E-09	ENSG0000	FLOT2/GA	139	-8.5046
BP	GO:00025	leukocyte	0.045228	470/16305	4.81E-08	4.57E-06	ENSG0000	CD109/GA	127	-5.34041
BP	GO:00509	leukocyte	0.043803	398/16305	9.1E-12	1.91E-09	ENSG0000	RPS19/GA	123	-8.71842
BP	GO:00027	immune re	0.042735	379/16305	2.73E-12	6.18E-10	ENSG0000	FCGR3A/H	120	-9.20888
BP	GO:00313	positive re	0.042379	455/16305	8.06E-07	5.65E-05	ENSG0000	RPS19/VA	119	-4.24791
BP	GO:00022	adaptive ir	0.042023	350/16305	3.32E-14	1.15E-11	ENSG0000	TGFB1/EBI	118	-10.9399
BP	GO:00512	regulation	0.042023	403/16305	9.89E-10	1.29E-07	ENSG0000	FLOT2/GA	118	-6.88812
BP	GO:00060	generation	0.042023	490/16305	5.61E-05	0.001976	ENSG0000	SH3BGRL3	118	-2.70414
BP	GO:00096	response t	0.041667	495/16305	0.000138	0.00415	ENSG0000	TSPO/RPS	117	-2.38191
BP	GO:00024	immune re	0.040598	347/16305	6.49E-13	1.78E-10	ENSG0000	FCGR3A/H	114	-9.75018
BP	GO:00457	positive re	0.040598	379/16305	3.3E-10	5.1E-08	ENSG0000	FLOT2/IBS	114	-7.29236
BP	GO:00508	regulation	0.040242	479/16305	0.000193	0.005529	ENSG0000	SLC29A1/I	113	-2.25737
BP	GO:00430	extracellul	0.039886	390/16305	8.73E-09	9.7E-07	ENSG0000	COMP/ITG	112	-6.01315
BP	GO:00450	regulation	0.039174	416/16305	1.19E-06	7.77E-05	ENSG0000	RPS19/MN	110	-4.10963
BP	GO:00018	positive re	0.038818	401/16305	3.1E-07	2.5E-05	ENSG0000	HPSE/TGF	109	-4.60165
BP	GO:00436	post-trans	0.038818	434/16305	1.65E-05	0.000702	ENSG0000	P4HB/QSC	109	-3.15352
BP	GO:00066	protein tar	0.038462	418/16305	4.69E-06	0.00024	ENSG0000	TSPO/RPL	108	-3.61935
BP	GO:00224	regulation	0.037749	373/16305	3.97E-08	3.83E-06	ENSG0000	FLOT2/TG	106	-5.41687
BP	GO:00064	RNA catab	0.037749	380/16305	1.12E-07	1.01E-05	ENSG0000	RPL18/RPI	106	-4.99424
BP	GO:00015	skeletal sy	0.037749	478/16305	0.002795	0.041595	ENSG0000	COMP/PP	106	-1.38096
BP	GO:00071	leukocyte	0.037393	321/16305	7.17E-12	1.56E-09	ENSG0000	FLOT2/TG	105	-8.80594
BP	GO:00091	glycoprote	0.037037	407/16305	1.17E-05	0.000522	ENSG0000	HPSE/CHF	104	-3.28251
BP	GO:00508	positive re	0.035969	308/16305	1.53E-11	2.9E-09	ENSG0000	FLOT2/GA	101	-8.53743
BP	GO:00064	mRNA cat	0.035969	347/16305	2.22E-08	2.25E-06	ENSG0000	RPL18/RPI	101	-5.64792
BP	GO:00508	regulation	0.035613	310/16305	5.58E-11	9.66E-09	ENSG0000	FLOT2/TG	100	-8.01506
BP	GO:00301	extracellul	0.035613	336/16305	7.41E-09	8.72E-07	ENSG0000	COMP/ITG	100	-6.05931
BP	GO:00026	positive re	0.035256	298/16305	1.04E-11	2.04E-09	ENSG0000	FLOT2/GA	99	-8.69066
BP	GO:00026	regulation	0.035256	338/16305	2.16E-08	2.23E-06	ENSG0000	RPS19/VA	99	-5.65216

BP	GO:00510	negative r	0.035256	433/1630	0.001402	0.025483	ENSG0000	ARL6IP5/T	99	-1.59376
BP	GO:00525	regulation	0.0349	404/1630	0.000175	0.005193	ENSG0000	ARL6IP5/C	98	-2.28461
BP	GO:00344	ncRNA pro	0.0349	418/1630	0.00062	0.013579	ENSG0000	RPL18/RPI	98	-1.86715
BP	GO:19019	regulation	0.034188	428/1630	0.003026	0.043082	ENSG0000	CDKN2A/T	96	-1.3657
BP	GO:00901	establishm	0.033832	297/1630	2.81E-10	4.47E-08	ENSG0000	YWHAH/R	95	-7.34968
BP	GO:19030	regulation	0.033476	289/1630	1.27E-10	2.14E-08	ENSG0000	FLOT2/TG	94	-7.66918
BP	GO:00069	response t	0.033476	419/1630	0.0033	0.046039	ENSG0000	ARL6IP5/P	94	-1.33687
BP	GO:00450	positive re	0.03312	351/1630	7.11E-06	0.000346	ENSG0000	RPS19/MN	93	-3.46118
BP	GO:19019	regulation	0.032764	395/1630	0.001114	0.020952	ENSG0000	CDKN2A/T	92	-1.67878
BP	GO:00525	regulation	0.032407	378/1630	0.000383	0.009684	ENSG0000	ARL6IP5/C	91	-2.01392
BP	GO:00198	antigen pr	0.032051	217/1630	2.48E-17	1.33E-14	ENSG0000	ITGB5/CTS	90	-13.8765
BP	GO:00704	response t	0.031695	371/1630	0.000499	0.011425	ENSG0000	SLC29A1/I	89	-1.94215
BP	GO:00300	lymphocyt	0.031339	320/1630	2.49E-06	0.000142	ENSG0000	GAS6/TGF	88	-3.84643
BP	GO:00422	ribosome	0.031339	330/1630	9.47E-06	0.000446	ENSG0000	RPL18/RPI	88	-3.35081
BP	GO:00512	positive re	0.030983	262/1630	1.9E-10	3.11E-08	ENSG0000	FLOT2/GA	87	-7.50704
BP	GO:00075	hemostasi	0.030983	334/1630	2.77E-05	0.00108	ENSG0000	HPSE/BLO	87	-2.96676
BP	GO:00069	phagocyto	0.030271	236/1630	2.48E-12	5.84E-10	ENSG0000	FCGR3A/G	85	-9.23355
BP	GO:00346	response t	0.030271	298/1630	7.26E-07	5.15E-05	ENSG0000	TMSB4X/T	85	-4.2883
BP	GO:00075	blood coa	0.030271	329/1630	4.67E-05	0.00172	ENSG0000	HPSE/BLO	85	-2.76448
BP	GO:00508	coagulatic	0.030271	331/1630	5.89E-05	0.002065	ENSG0000	HPSE/BLO	85	-2.68507
BP	GO:00022	response t	0.030271	336/1630	0.000103	0.003379	ENSG0000	TSPO/TGF	85	-2.47127
BP	GO:00706	leukocyte	0.029915	268/1630	8.71E-09	9.7E-07	ENSG0000	TGFB1/EBI	84	-6.01315
BP	GO:00507	regulation	0.029558	332/1630	0.000189	0.0055	ENSG0000	TMSB4X/R	83	-2.25963
BP	GO:00362	response t	0.029558	348/1630	0.00091	0.018105	ENSG0000	SLC29A1/I	83	-1.7422
BP	GO:00224	positive re	0.029202	245/1630	4.09E-10	5.94E-08	ENSG0000	FLOT2/TG	82	-7.2259
BP	GO:00160	rRNA meta	0.029202	299/1630	6.06E-06	0.0003	ENSG0000	RPL18/RPI	82	-3.52288
BP	GO:00516	protein ma	0.029202	322/1630	0.000107	0.003478	ENSG0000	LMF2/CTS	82	-2.45869
BP	GO:00016	response t	0.028846	340/1630	0.001079	0.020488	ENSG0000	SLC29A1/I	81	-1.6885
BP	GO:00064	translatior	0.02849	193/1630	1.53E-15	6.93E-13	ENSG0000	RPL18/RPI	80	-12.1591
BP	GO:19030	positive re	0.02849	212/1630	6.64E-13	1.78E-10	ENSG0000	FLOT2/TG	80	-9.75018
BP	GO:00713	cellular res	0.02849	281/1630	1.65E-06	0.0001	ENSG0000	TMSB4X/T	80	-3.99902
BP	GO:00022	activation	0.02849	299/1630	2.08E-05	0.000856	ENSG0000	CTSB/NOF	80	-3.06729
BP	GO:00091	glycoprote	0.02849	338/1630	0.001406	0.025483	ENSG0000	CHPF2/DD	80	-1.59376
BP	GO:00508	antigen re	0.028134	232/1630	3.38E-10	5.1E-08	ENSG0000	HLA-DQB	79	-7.29236
BP	GO:00466	lymphocyt	0.028134	250/1630	1.61E-08	1.72E-06	ENSG0000	TGFB1/EBI	79	-5.76448
BP	GO:00329	mononucl	0.028134	252/1630	2.37E-08	2.37E-06	ENSG0000	TGFB1/EBI	79	-5.62545

BP	GO:00324	response t	0.028134	318/16305	0.000328	0.008535	ENSG0000(	TSPO/TGF	79	-2.06879
BP	GO:00480	antigen pr	0.027778	183/16305	5.33E-16	2.61E-13	ENSG0000(	ITGB5/CTS	78	-12.5827
BP	GO:00508	positive re	0.027422	203/16305	1.32E-12	3.24E-10	ENSG0000(	FLOT2/TG	77	-9.48985
BP	GO:00603	cell chemo	0.027422	257/16305	2.86E-07	2.38E-05	ENSG0000(	TMSB4X/R	77	-4.62427
BP	GO:00027	innate imr	0.027422	280/16305	1.03E-05	0.000472	ENSG0000(	CTSB/NOF	77	-3.32644
BP	GO:00329	regulation	0.027422	320/16305	0.001048	0.020337	ENSG0000(	PFN1/TMS	77	-1.69171
BP	GO:00091	nucleoside	0.027422	332/16305	0.00303	0.043082	ENSG0000(	TSPO/TMS	77	-1.3657
BP	GO:00063	rRNA proc	0.027066	265/16305	2.2E-06	0.000127	ENSG0000(	RPL18/RPI	76	-3.89575
BP	GO:00466	response t	0.027066	311/16305	0.000705	0.015042	ENSG0000(	SDF4/CST3	76	-1.8227
BP	GO:00190	viral life cy	0.027066	316/16305	0.001142	0.021273	ENSG0000(	ITGB5/P4H	76	-1.67217
BP	GO:00709	protein loc	0.026353	131/16305	2.62E-24	1.68E-21	ENSG0000(	RPL18/RPI	74	-20.7756
BP	GO:00066	protein tar	0.026353	183/16305	8.16E-14	2.67E-11	ENSG0000(	RPL18/ICM	74	-10.5736
BP	GO:00190	viral gene	0.026353	190/16305	7.76E-13	1.99E-10	ENSG0000(	RPL18/RPI	74	-9.70173
BP	GO:00512	maintenar	0.026353	305/16305	0.001018	0.019914	ENSG0000(	TSPO/TMS	74	-1.70083
BP	GO:00329	protein-co	0.026353	312/16305	0.001956	0.032158	ENSG0000(	CAPG/GAI	74	-1.49271
BP	GO:00198	antigen pr	0.025641	177/16305	1.24E-13	3.84E-11	ENSG0000(	ITGB5/CTS	72	-10.4158
BP	GO:00349	response t	0.025641	271/16305	6.7E-05	0.002322	ENSG0000(	P4HB/MYI	72	-2.63418
BP	GO:00513	negative r	0.025641	299/16305	0.001464	0.02587	ENSG0000(	PODNL1/(	72	-1.5872
BP	GO:00009	nuclear-tr	0.025285	209/16305	2.94E-09	3.68E-07	ENSG0000(	RPL18/RPI	71	-6.43402
BP	GO:00072	I-kappaB I	0.025285	253/16305	1.07E-05	0.00048	ENSG0000(	RHOA/AKT	71	-3.31876
BP	GO:00024	antigen pr	0.024929	170/16305	1.33E-13	3.91E-11	ENSG0000(	ITGB5/CTS	70	-10.4084
BP	GO:00321	positive re	0.024573	279/16305	0.00086	0.017405	ENSG0000(	TMSB4X/R	69	-1.75934
BP	GO:00190	viral transc	0.024217	176/16305	1.01E-11	2.04E-09	ENSG0000(	RPL18/RPI	68	-8.69066
BP	GO:00024	adaptive ir	0.024217	218/16305	2.71E-07	2.28E-05	ENSG0000(	TGFB1/EBI	68	-4.6422
BP	GO:00024	lymphocyt	0.024217	219/16305	3.28E-07	2.61E-05	ENSG0000(	VAMP7/T(	68	-4.58371
BP	GO:19011	carbohydr	0.023504	184/16305	8.34E-10	1.12E-07	ENSG0000(	HPSE/CST	66	-6.95255
BP	GO:00508	T cell rece	0.023504	193/16305	7.77E-09	8.96E-07	ENSG0000(	HLA-DQB	66	-6.04747
BP	GO:01400	mitotic nu	0.023504	265/16305	0.000905	0.018055	ENSG0000(	TGFB1/FBX	66	-1.7434
BP	GO:00725	establishm	0.023148	106/16305	2.85E-24	1.68E-21	ENSG0000(	RPL18/RPI	65	-20.7756
BP	GO:00706	regulation	0.023148	210/16305	6.7E-07	4.87E-05	ENSG0000(	TGFB1/EBI	65	-4.31276
BP	GO:00160	carbohydr	0.023148	216/16305	1.99E-06	0.000117	ENSG0000(	PGAM1/G	65	-3.93074
BP	GO:00068	receptor-r	0.023148	272/16305	0.002929	0.042166	ENSG0000(	CAP1/RAE	65	-1.37504
BP	GO:00450	protein tar	0.022792	102/16305	1.01E-24	9.95E-22	ENSG0000(	RPL18/RPI	64	-21.0021
BP	GO:00506	regulation	0.022792	199/16305	1.77E-07	1.53E-05	ENSG0000(	TGFB1/EBI	64	-4.81532
BP	GO:00329	regulation	0.022792	200/16305	2.17E-07	1.85E-05	ENSG0000(	TGFB1/EBI	64	-4.73298
BP	GO:20000	regulation	0.022792	242/16305	0.00019	0.005522	ENSG0000(	PFN1/TGF	64	-2.25794

BP	GO:00164	protein pr	0.022792	260/16305	0.001468	0.02587	ENSG0000(	CTSZ/GAS	64	-1.5872
BP	GO:00700	glycosylati	0.022792	268/16305	0.003192	0.04485	ENSG0000(	DDOST/D/	64	-1.34824
BP	GO:00420	T cell proli	0.022436	172/16305	7.6E-10	1.04E-07	ENSG0000(	TGFB1/EBI	63	-6.98287
BP	GO:00302	T cell diffe	0.022436	223/16305	2.62E-05	0.001031	ENSG0000(	TGFB1/RH	63	-2.98689
BP	GO:00064	protein gly	0.022436	260/16305	0.002382	0.036612	ENSG0000(	DDOST/D/	63	-1.43637
BP	GO:00434	macromol	0.022436	260/16305	0.002382	0.036612	ENSG0000(	DDOST/D/	63	-1.43637
BP	GO:00066	cotranslati	0.02208	97/16305	1.25E-24	1.05E-21	ENSG0000(	RPL18/RPI	62	-20.978
BP	GO:00431	regulation	0.02208	220/16305	3.28E-05	0.001252	ENSG0000(	RHOA/AKT	62	-2.90236
BP	GO:20001	regulation	0.02208	235/16305	0.000255	0.007041	ENSG0000(	ARL6IP5/G	62	-2.15239
BP	GO:19019	negative r	0.02208	239/16305	0.000416	0.010198	ENSG0000(	TRIAP1/RF	62	-1.99149
BP	GO:00305	leukocyte	0.021724	195/16305	9.82E-07	6.58E-05	ENSG0000(	RPS19/GA	61	-4.18183
BP	GO:00066	SRP-depe	0.021368	92/16305	1.45E-24	1.07E-21	ENSG0000(	RPL18/RPI	60	-20.972
BP	GO:00060	aminoglyc	0.021011	171/16305	3.3E-08	3.24E-06	ENSG0000(	HPSE/HS3	59	-5.48941
BP	GO:00018	negative r	0.021011	240/16305	0.002264	0.03631	ENSG0000(	TSPO/TMS	59	-1.43998
BP	GO:00001	nuclear-tr	0.020655	121/16305	5.5E-15	2.16E-12	ENSG0000(	RPL18/RPI	58	-11.6657
BP	GO:00421	B cell activ	0.020655	221/16305	0.000458	0.010851	ENSG0000(	TGFB1/CD	58	-1.96455
BP	GO:19019	negative r	0.020655	221/16305	0.000458	0.010851	ENSG0000(	TRIAP1/RF	58	-1.96455
BP	GO:00301	regulation	0.020655	227/16305	0.000934	0.018451	ENSG0000(	PPT1/GAS	58	-1.73397
BP	GO:00008	sister chro	0.020655	238/16305	0.003019	0.043082	ENSG0000(	RPS27L/PI	58	-1.3657
BP	GO:00380	Fc recepto	0.020299	187/16305	5.38E-06	0.000273	ENSG0000(	FCGR3A/U	57	-3.56374
BP	GO:00332	tumor nec	0.019943	170/16305	4.24E-07	3.32E-05	ENSG0000(	TMSB4X/T	56	-4.47899
BP	GO:00343	response t	0.019943	186/16305	9.74E-06	0.000455	ENSG0000(	RPL13A/H	56	-3.34176
BP	GO:00712	cellular res	0.019943	197/16305	5.94E-05	0.002069	ENSG0000(	TSPO/TGF	56	-2.68417
BP	GO:00432	regulation	0.019943	210/16305	0.00037	0.00948	ENSG0000(	ARL6IP5/G	56	-2.02318
BP	GO:00431	positive re	0.019587	166/16305	4.34E-07	3.32E-05	ENSG0000(	RHOA/AKT	55	-4.47899
BP	GO:00517	interactior	0.019587	209/16305	0.000589	0.013043	ENSG0000(	ITGB5/P4H	55	-1.88462
BP	GO:00714	cellular res	0.019587	216/16305	0.001356	0.024863	ENSG0000(	SLC29A1/I	55	-1.60445
BP	GO:00972	cellular res	0.019587	217/16305	0.001517	0.026667	ENSG0000(	CST3/PSA	55	-1.57402
BP	GO:00027	regulation	0.019231	167/16305	1.3E-06	8.22E-05	ENSG0000(	VAMP7/TC	54	-4.08528
BP	GO:00026	regulation	0.019231	171/16305	2.93E-06	0.000161	ENSG0000(	GAS6/TGF	54	-3.79295
BP	GO:00081	actin polyr	0.019231	198/16305	0.000256	0.007041	ENSG0000(	PFN1/CAP	54	-2.15239
BP	GO:00436	cellular pr	0.019231	207/16305	0.000821	0.016951	ENSG0000(	CAPG/GAI	54	-1.7708
BP	GO:00713	cellular res	0.018875	167/16305	3.05E-06	0.000166	ENSG0000(	RPL13A/H	53	-3.77881
BP	GO:00514	positive re	0.018875	211/16305	0.002252	0.036227	ENSG0000(	PFN1/RHC	53	-1.44097
BP	GO:00421	regulation	0.018519	147/16305	8.45E-08	7.77E-06	ENSG0000(	TGFB1/EBI	52	-5.10967
BP	GO:00026	positive re	0.018519	173/16305	2.09E-05	0.000856	ENSG0000(	RPS19/VA	52	-3.06729

BP	GO:00712	cellular res	0.018519	175/16305	2.96E-05	0.001148	ENSG0000	TSPO/TGF	52	-2.94016
BP	GO:00506	cytokine s	0.018519	189/16305	0.000261	0.007153	ENSG0000	TMSB4X/G	52	-2.14554
BP	GO:00301	platelet ac	0.018162	156/16305	1.76E-06	0.000105	ENSG0000	BLOC1S4/	51	-3.97975
BP	GO:00302	glycosami	0.018162	158/16305	2.68E-06	0.000151	ENSG0000	HPSE/HS3	51	-3.8224
BP	GO:19029	positive re	0.018162	189/16305	0.00049	0.011392	ENSG0000	PFN1/COL	51	-1.94339
BP	GO:00362	cellular res	0.018162	201/16305	0.002147	0.034913	ENSG0000	SLC29A1/I	51	-1.45702
BP	GO:00433	regulation	0.018162	201/16305	0.002147	0.034913	ENSG0000	CTSZ/AKT	51	-1.45702
BP	GO:00229	electron tr	0.017806	175/16305	0.000125	0.003942	ENSG0000	SH3BGRL3	50	-2.40428
BP	GO:00975	myeloid le	0.01745	170/16305	0.000115	0.003698	ENSG0000	RPS19/CM	49	-2.43203
BP	GO:00022	pattern re	0.01745	181/16305	0.000578	0.012881	ENSG0000	CTSB/NOF	49	-1.89005
BP	GO:00714	cellular res	0.01745	194/16305	0.002858	0.042144	ENSG0000	SLC29A1/I	49	-1.37527
BP	GO:00080	regulation	0.017094	176/16305	0.000547	0.012233	ENSG0000	PFN1/TMS	48	-1.91245
BP	GO:00313	negative r	0.017094	176/16305	0.000547	0.012233	ENSG0000	RPS19/AC	48	-1.91245
BP	GO:00308	regulation	0.017094	177/16305	0.000627	0.013666	ENSG0000	PFN1/TMS	48	-1.86437
BP	GO:19053	regulation	0.017094	178/16305	0.000717	0.015245	ENSG0000	PFN1/TGF	48	-1.81688
BP	GO:00603	bone deve	0.017094	184/16305	0.001547	0.027017	ENSG0000	COMP/PP	48	-1.56836
BP	GO:00712	cellular res	0.016738	167/16305	0.000288	0.007742	ENSG0000	TSPO/TGF	47	-2.11116
BP	GO:00508	negative r	0.016738	170/16305	0.000447	0.010694	ENSG0000	TGFB1/TN	47	-1.97084
BP	GO:00300	actin filam	0.016738	171/16305	0.000515	0.011708	ENSG0000	PFN1/TMS	47	-1.93152
BP	GO:00069	response t	0.016738	178/16305	0.001313	0.02423	ENSG0000	MYDGF/PI	47	-1.61565
BP	GO:00022	toll-like re	0.016026	135/16305	3.92E-06	0.000204	ENSG0000	CTSB/UBA	45	-3.6901
BP	GO:00706	positive re	0.016026	138/16305	7.53E-06	0.000363	ENSG0000	EBI3/HLA-	45	-3.43963
BP	GO:00000	mitotic sis	0.016026	151/16305	9.14E-05	0.003058	ENSG0000	PSMG2/CH	45	-2.51463
BP	GO:00024	productio	0.016026	167/16305	0.00105	0.020337	ENSG0000	GAS6/TGF	45	-1.69171
BP	GO:00024	antigen pr	0.01567	93/16305	1.81E-11	3.23E-09	ENSG0000	ITGB5/PSM	44	-8.49105
BP	GO:00506	positive re	0.01567	130/16305	3.17E-06	0.000171	ENSG0000	EBI3/HLA-	44	-3.76674
BP	GO:00329	positive re	0.01567	131/16305	3.98E-06	0.000206	ENSG0000	EBI3/HLA-	44	-3.68672
BP	GO:00026	positive re	0.015313	123/16305	1.55E-06	9.6E-05	ENSG0000	GAS6/TGF	43	-4.01765
BP	GO:00019	cell killing	0.015313	125/16305	2.52E-06	0.000143	ENSG0000	RPS19/VA	43	-3.84544
BP	GO:00308	regulation	0.015313	157/16305	0.00094	0.018511	ENSG0000	PFN1/TMS	43	-1.73256
BP	GO:00507	regulation	0.015313	165/16305	0.002686	0.04023	ENSG0000	TMSB4X/G	43	-1.39545
BP	GO:00329	collagen m	0.014957	116/16305	7.04E-07	5.05E-05	ENSG0000	CTSD/FAP	42	-4.29657
BP	GO:00025	platelet de	0.014957	127/16305	1.01E-05	0.000466	ENSG0000	TMSB4X/Q	42	-3.33203
BP	GO:00064	translatior	0.014957	131/16305	2.37E-05	0.000954	ENSG0000	RPLP1/GA	42	-3.02034
BP	GO:00302	entry into	0.014957	135/16305	5.23E-05	0.001854	ENSG0000	ITGB5/P4H	42	-2.73178
BP	GO:00444	entry into	0.014957	135/16305	5.23E-05	0.001854	ENSG0000	ITGB5/P4H	42	-2.73178

BP	GO:00518	entry into	0.014957	135/16305	5.23E-05	0.001854	ENSG0000	ITGB5/P4H	42	-2.73178
BP	GO:00518	entry into	0.014957	135/16305	5.23E-05	0.001854	ENSG0000	ITGB5/P4H	42	-2.73178
BP	GO:00487	tissue rem	0.014601	151/16305	0.00146	0.02587	ENSG0000	CST3/TGF	41	-1.5872
BP	GO:00022	lymphocyt	0.014601	153/16305	0.001912	0.031801	ENSG0000	VAMP7/TC	41	-1.49756
BP	GO:00421	positive re	0.014245	94/16305	7.18E-09	8.63E-07	ENSG0000	EBI3/HLA-	40	-6.06404
BP	GO:00066	glycolipid	0.014245	121/16305	1.65E-05	0.000702	ENSG0000	PSAP/PIGK	40	-3.15352
BP	GO:19035	liposaccha	0.014245	121/16305	1.65E-05	0.000702	ENSG0000	PSAP/PIGK	40	-3.15352
BP	GO:00507	negative r	0.014245	137/16305	0.000349	0.009013	ENSG0000	RPS19/TG	40	-2.04515
BP	GO:19026	proton tra	0.014245	143/16305	0.000894	0.017896	ENSG0000	TMSB4X/U	40	-1.74723
BP	GO:00355	non-cano	0.014245	153/16305	0.00348	0.048197	ENSG0000	PFN1/RHO	40	-1.31698
BP	GO:00326	interferon	0.013533	100/16305	5.83E-07	4.4E-05	ENSG0000	GAS6/EBI3	38	-4.35639
BP	GO:00060	aminoglyc	0.013533	112/16305	1.36E-05	0.000597	ENSG0000	HS3ST2/C	38	-3.22393
BP	GO:00380	NIK/NF-ka	0.013533	123/16305	0.000137	0.00415	ENSG0000	TMSB4X/A	38	-2.38191
BP	GO:00325	mitochond	0.013533	130/16305	0.000473	0.011129	ENSG0000	GADD45G	38	-1.95353
BP	GO:00425	antigen pr	0.013177	77/16305	4.14E-10	5.94E-08	ENSG0000	ITGB5/PSM	37	-7.2259
BP	GO:00024	antigen pr	0.013177	98/16305	9.83E-07	6.58E-05	ENSG0000	CTSD/HLA	37	-4.18183
BP	GO:00025	antigen pr	0.013177	99/16305	1.31E-06	8.22E-05	ENSG0000	CTSD/HLA	37	-4.08528
BP	GO:00718	leukocyte	0.013177	100/16305	1.74E-06	0.000105	ENSG0000	CDKN2A/C	37	-3.98075
BP	GO:00467	viral entry	0.013177	119/16305	0.000144	0.004303	ENSG0000	ITGB5/P4H	37	-2.36626
BP	GO:00509	positive re	0.013177	130/16305	0.000965	0.018936	ENSG0000	TMSB4X/C	37	-1.72271
BP	GO:00712	cellular res	0.013177	131/16305	0.001126	0.021103	ENSG0000	CST3/PSA	37	-1.67566
BP	GO:00024	antigen pr	0.012821	74/16305	4.61E-10	6.46E-08	ENSG0000	ITGB5/PSM	36	-7.19009
BP	GO:00064	translation	0.012821	102/16305	8.36E-06	0.000397	ENSG0000	GADD45G	36	-3.40145
BP	GO:00027	regulation	0.012821	123/16305	0.00064	0.013897	ENSG0000	TGFB1/TN	36	-1.85708
BP	GO:00028	regulation	0.012821	130/16305	0.001902	0.031757	ENSG0000	TGFB1/TN	36	-1.49817
BP	GO:00093	amine me	0.012821	132/16305	0.002529	0.038471	ENSG0000	SRM/OAZ	36	-1.41486
BP	GO:19021	positive re	0.012821	132/16305	0.002529	0.038471	ENSG0000	GAS6/TGF	36	-1.41486
BP	GO:00019	leukocyte	0.012464	89/16305	6.21E-07	4.58E-05	ENSG0000	VAMP7/FA	35	-4.33905
BP	GO:00726	lymphocyt	0.012464	90/16305	8.5E-07	5.82E-05	ENSG0000	GAS6/RHO	35	-4.23531
BP	GO:00060	glycosami	0.012464	107/16305	6.92E-05	0.002384	ENSG0000	HS3ST2/C	35	-2.62278
BP	GO:00198	antigen pr	0.012108	95/16305	1.05E-05	0.000473	ENSG0000	CTSD/HLA	34	-3.32484
BP	GO:00600	Wnt signa	0.012108	110/16305	0.000297	0.007925	ENSG0000	PFN1/RHO	34	-2.10102
BP	GO:00901	regulation	0.012108	112/16305	0.000431	0.010405	ENSG0000	PFN1/RHO	34	-1.98277
BP	GO:00027	regulation	0.012108	114/16305	0.000616	0.013522	ENSG0000	TGFB1/TN	34	-1.86895
BP	GO:19035	mucopoly	0.012108	115/16305	0.000732	0.015439	ENSG0000	CHPF2/CH	34	-1.81139
BP	GO:19035	regulation	0.012108	115/16305	0.000732	0.015439	ENSG0000	TSPO/GAS	34	-1.81139

BP	GO:00975	granulocy	0.012108	117/16305	0.001023	0.019939	ENSG0000	CMKLR1/II	34	-1.7003
BP	GO:00717	tumor nec	0.012108	119/16305	0.001411	0.025483	ENSG0000	TSPO/GAS	34	-1.59376
BP	GO:00017	establishm	0.012108	122/16305	0.00223	0.036067	ENSG0000	PFN1/RHC	34	-1.44289
BP	GO:00071	establishm	0.012108	122/16305	0.00223	0.036067	ENSG0000	PFN1/RHC	34	-1.44289
BP	GO:00441	cellular an	0.012108	123/16305	0.002582	0.039173	ENSG0000	SRM/OAZ	34	-1.40701
BP	GO:00024	Fc recepto	0.011752	78/16305	1.72E-07	1.51E-05	ENSG0000	FCGR3A/A	33	-4.82008
BP	GO:00701	mitochond	0.011752	87/16305	3.31E-06	0.000177	ENSG0000	GADD45G	33	-3.75186
BP	GO:00701	mitochond	0.011752	89/16305	5.88E-06	0.000293	ENSG0000	GADD45G	33	-3.53251
BP	GO:00072	integrin-m	0.011752	97/16305	4.6E-05	0.001702	ENSG0000	ITGB5/ITG	33	-2.76906
BP	GO:00070	mitotic spi	0.011752	112/16305	0.000927	0.018379	ENSG0000	MZT1/RH(	33	-1.73567
BP	GO:00028	regulation	0.011752	119/16305	0.002798	0.041595	ENSG0000	TGFB1/TN	33	-1.38096
BP	GO:00060	aminoglyc	0.011396	69/16305	1.85E-08	1.95E-06	ENSG0000	HPSE/TGF	32	-5.7103
BP	GO:00341	homotypic	0.011396	74/16305	1.43E-07	1.28E-05	ENSG0000	BLOC1S4/	32	-4.89415
BP	GO:00380	Fc-gamma	0.011396	77/16305	4.31E-07	3.32E-05	ENSG0000	FCGR3A/A	32	-4.47899
BP	GO:00021	cytoplasm	0.011396	87/16305	9.84E-06	0.000456	ENSG0000	RPL18/RPL	32	-3.34106
BP	GO:00326	regulation	0.011396	89/16305	1.69E-05	0.000715	ENSG0000	GAS6/EBI3	32	-3.14563
BP	GO:19027	negative r	0.011396	105/16305	0.000581	0.012903	ENSG0000	NOP53/UE	32	-1.88929
BP	GO:00326	regulation	0.011396	111/16305	0.001634	0.028044	ENSG0000	TSPO/GAS	32	-1.55216
BP	GO:00466	alpha-beta	0.011396	113/16305	0.002241	0.036137	ENSG0000	EBI3/RHO/	32	-1.44205
BP	GO:00326	tumor nec	0.011396	114/16305	0.00261	0.039504	ENSG0000	TSPO/GAS	32	-1.40336
BP	GO:00103	membrane	0.01104	63/16305	5.2E-09	6.38E-07	ENSG0000	VAMP7/RI	31	-6.19507
BP	GO:00226	extracellul	0.01104	92/16305	9.46E-05	0.003112	ENSG0000	CAPNS1/F	31	-2.50695
BP	GO:00308	positive re	0.01104	97/16305	0.000281	0.007576	ENSG0000	PFN1/RHC	31	-2.12056
BP	GO:00026	negative r	0.01104	104/16305	0.001057	0.02041	ENSG0000	RPS19/TG	31	-1.69016
BP	GO:00026	regulation	0.01104	109/16305	0.002418	0.036973	ENSG0000	GAS6/CMI	31	-1.43212
BP	GO:00024	immune re	0.010684	73/16305	1.3E-06	8.22E-05	ENSG0000	FCGR3A/A	30	-4.08528
BP	GO:00380	Fc-gamma	0.010684	73/16305	1.3E-06	8.22E-05	ENSG0000	FCGR3A/A	30	-4.08528
BP	GO:00311	anaphase-	0.010684	80/16305	1.19E-05	0.000525	ENSG0000	UBA52/PS	30	-3.27955
BP	GO:20001	regulation	0.010684	82/16305	2.08E-05	0.000856	ENSG0000	CDKN2A/(	30	-3.06729
BP	GO:00990	plasma me	0.010328	55/16305	2.06E-09	2.64E-07	ENSG0000	VAMP7/RI	29	-6.57901
BP	GO:00454	cell redox	0.010328	70/16305	1.6E-06	9.8E-05	ENSG0000	P4HB/QSC	29	-4.00863
BP	GO:20001	negative r	0.010328	97/16305	0.001433	0.025483	ENSG0000	GAS6/CST	29	-1.59376
BP	GO:00705	platelet ag	0.009972	55/16305	1.09E-08	1.19E-06	ENSG0000	BLOC1S4/	28	-5.92538
BP	GO:00702	lymphocyt	0.009972	68/16305	2.78E-06	0.000155	ENSG0000	AKT1/CD7	28	-3.81099
BP	GO:00312	T cell costi	0.009972	69/16305	3.92E-06	0.000204	ENSG0000	AKT1/HLA	28	-3.6901
BP	GO:00312	lymphocyt	0.009972	70/16305	5.46E-06	0.000275	ENSG0000	AKT1/HLA	28	-3.56074

BP	GO:00093	oligosacch	0.009972	76/16305	3.35E-05	0.001271	ENSG0000	GALNT5/C	28	-2.89573
BP	GO:00603	interferon	0.009972	86/16305	0.000386	0.009706	ENSG0000	HLA-DQB	28	-2.01297
BP	GO:00064	protein N-	0.009972	90/16305	0.000872	0.017514	ENSG0000	DDOST/DA	28	-1.75662
BP	GO:00420	regulation	0.009972	94/16305	0.001822	0.031094	ENSG0000	EBI3/CD27	28	-1.50732
BP	GO:00434	pigmentat	0.009972	97/16305	0.003025	0.043082	ENSG0000	BLOC1S4/	28	-1.3657
BP	GO:00060	glycosami	0.009615	62/16305	1.13E-06	7.44E-05	ENSG0000	HPSE/TGF	27	-4.1282
BP	GO:00305	collagen c	0.009615	65/16305	3.41E-06	0.000181	ENSG0000	CTSD/FAP	27	-3.74208
BP	GO:00332	regulation	0.009615	77/16305	0.000123	0.003884	ENSG0000	OAZ1/PSM	27	-2.4107
BP	GO:00026	positive re	0.009615	87/16305	0.001114	0.020952	ENSG0000	GAS6/CMI	27	-1.67878
BP	GO:20003	positive re	0.009615	87/16305	0.001114	0.020952	ENSG0000	TSPO/TGF	27	-1.67878
BP	GO:00109	negative r	0.009615	93/16305	0.003254	0.045498	ENSG0000	NOP53/UB	27	-1.34201
BP	GO:00480	vascular e	0.009615	93/16305	0.003254	0.045498	ENSG0000	RHOA/VEG	27	-1.34201
BP	GO:00324	lysosome	0.009259	66/16305	1.62E-05	0.000702	ENSG0000	VAMP7/H	26	-3.15352
BP	GO:00066	glycosphir	0.009259	72/16305	9.32E-05	0.003099	ENSG0000	PSAP/CTS	26	-2.50873
BP	GO:00507	regulation	0.009259	73/16305	0.000121	0.00386	ENSG0000	GAS6/TGF	26	-2.41336
BP	GO:00716	mononucl	0.009259	75/16305	0.000201	0.005745	ENSG0000	RPS19/TG	26	-2.24074
BP	GO:00024	T cell med	0.009259	81/16305	0.000781	0.016191	ENSG0000	FADD/TRF	26	-1.79074
BP	GO:00346	cellular ca	0.009259	81/16305	0.000781	0.016191	ENSG0000	AKT1/AKR	26	-1.79074
BP	GO:00017	leukocyte	0.009259	84/16305	0.001423	0.025483	ENSG0000	TGFB1/AK	26	-1.59376
BP	GO:00456	positive re	0.008903	83/16305	0.002638	0.039719	ENSG0000	GAS6/TGF	25	-1.401
BP	GO:00481	regulation	0.008903	83/16305	0.002638	0.039719	ENSG0000	GAS6/TGF	25	-1.401
BP	GO:00481	fibroblast	0.008903	84/16305	0.003153	0.044405	ENSG0000	GAS6/TGF	25	-1.35257
BP	GO:00069	phagocyto	0.008547	46/16305	6.61E-08	6.17E-06	ENSG0000	VAMP7/IT	24	-5.20954
BP	GO:00726	T cell migr	0.008547	49/16305	3.1E-07	2.5E-05	ENSG0000	RHOA/ECI	24	-4.60165
BP	GO:00070	lysosome	0.008547	60/16305	2.52E-05	0.001001	ENSG0000	PPT1/VPS3	24	-2.9996
BP	GO:00801	lytic vacuc	0.008547	60/16305	2.52E-05	0.001001	ENSG0000	PPT1/VPS3	24	-2.9996
BP	GO:00326	interleukir	0.008547	68/16305	0.000256	0.007041	ENSG0000	TMSB4X/F	24	-2.15239
BP	GO:00311	SCF-depe	0.008547	73/16305	0.000837	0.017204	ENSG0000	FBXL15/FB	24	-1.76437
BP	GO:00311	animal org	0.008547	76/16305	0.001573	0.027245	ENSG0000	UCP2/GAS	24	-1.56471
BP	GO:00327	positive re	0.008191	60/16305	8.12E-05	0.002746	ENSG0000	EBI3/HLA-	23	-2.56134
BP	GO:00022	lymphocyt	0.008191	63/16305	0.000192	0.005529	ENSG0000	TGFB1/AK	23	-2.25737
BP	GO:19035	positive re	0.008191	69/16305	0.000859	0.017405	ENSG0000	CD14/FAD	23	-1.75934
BP	GO:00313	regulation	0.008191	70/16305	0.001074	0.020454	ENSG0000	FADD/GAI	23	-1.68922
BP	GO:00002	polysaccha	0.008191	75/16305	0.002977	0.042739	ENSG0000	TGFB1/AK	23	-1.36918
BP	GO:00019	regulation	0.007835	58/16305	0.000138	0.00415	ENSG0000	FADD/LAM	22	-2.38191
BP	GO:00022	natural kill	0.007835	62/16305	0.000418	0.010213	ENSG0000	VAMP7/LA	22	-1.99085

BP	GO:00702	T cell apop	0.007479	46/16305	7.01E-06	0.000344	ENSG0000(	AKT1/FAD	21	-3.46354
BP	GO:00702	regulation	0.007479	53/16305	9.37E-05	0.003099	ENSG0000(	CD74/FAD	21	-2.50873
BP	GO:00068	iron ion tra	0.007479	57/16305	0.000312	0.008267	ENSG0000(	STEAP3/AT	21	-2.08266
BP	GO:00422	natural kill	0.007479	58/16305	0.00041	0.01014	ENSG0000(	VAMP7/LA	21	-1.99396
BP	GO:00065	regulation	0.007479	59/16305	0.000535	0.012063	ENSG0000(	OAZ1/PSM	21	-1.91855
BP	GO:00422	ribosome	0.007479	59/16305	0.000535	0.012063	ENSG0000(	RPS19/RPS	21	-1.91855
BP	GO:00327	positive re	0.007479	66/16305	0.002711	0.040501	ENSG0000(	CD14/FAD	21	-1.39253
BP	GO:00433	regulation	0.007123	43/16305	8.15E-06	0.00039	ENSG0000(	VAMP7/IT	20	-3.4088
BP	GO:00028	regulation	0.007123	45/16305	1.89E-05	0.00079	ENSG0000(	VAMP7/IT	20	-3.10229
BP	GO:00458	pH reduct	0.007123	50/16305	0.000117	0.003746	ENSG0000(	PPT1/ATP(	20	-2.42645
BP	GO:00508	B cell rece	0.007123	53/16305	0.000297	0.007925	ENSG0000(	BAX/NCKA	20	-2.10102
BP	GO:00455	mast cell a	0.007123	56/16305	0.000684	0.014668	ENSG0000(	VAMP7/M	20	-1.83362
BP	GO:00326	regulation	0.007123	61/16305	0.002278	0.03633	ENSG0000(	TMSB4X/F	20	-1.43974
BP	GO:00301	collagen fi	0.006766	44/16305	4.98E-05	0.00181	ENSG0000(	COLGALT1	19	-2.74242
BP	GO:00027	negative r	0.006766	45/16305	7.24E-05	0.002462	ENSG0000(	IL13RA2/N	19	-2.60867
BP	GO:00450	T cell selec	0.006766	45/16305	7.24E-05	0.002462	ENSG0000(	RHOA/CD	19	-2.60867
BP	GO:00514	intracellula	0.006766	49/16305	0.000278	0.00755	ENSG0000(	PPT1/ATP(	19	-2.12207
BP	GO:00451	cellular ex	0.006766	51/16305	0.000505	0.011523	ENSG0000(	FADD/ITG	19	-1.93844
BP	GO:00069	cellular de	0.006766	52/16305	0.000669	0.014421	ENSG0000(	TYROBP/T	19	-1.84102
BP	GO:00330	cellular pig	0.006766	52/16305	0.000669	0.014421	ENSG0000(	BLOC1S4/	19	-1.84102
BP	GO:00508	regulation	0.006766	54/16305	0.001136	0.021238	ENSG0000(	RAB29/KC	19	-1.67288
BP	GO:00421	positive re	0.006766	57/16305	0.002344	0.036585	ENSG0000(	EBI3/CD27	19	-1.4367
BP	GO:00970	dendritic c	0.00641	39/16305	2.63E-05	0.001031	ENSG0000(	GAS6/TGF	18	-2.98689
BP	GO:00903	phagosom	0.00641	42/16305	8.82E-05	0.002968	ENSG0000(	RAB31/RA	18	-2.52751
BP	GO:00182	protein N-	0.00641	43/16305	0.000127	0.003992	ENSG0000(	DDOST/DA	18	-2.39878
BP	GO:00022	mast cell a	0.00641	44/16305	0.000181	0.00531	ENSG0000(	VAMP7/M	18	-2.27494
BP	GO:00181	peptidyl-a	0.00641	44/16305	0.000181	0.00531	ENSG0000(	DDOST/DA	18	-2.27494
BP	GO:00433	mast cell c	0.00641	44/16305	0.000181	0.00531	ENSG0000(	VAMP7/M	18	-2.27494
BP	GO:00464	icosanoid	0.00641	45/16305	0.000254	0.007041	ENSG0000(	MGST3/CD	18	-2.15239
BP	GO:00024	mast cell r	0.00641	46/16305	0.000351	0.009013	ENSG0000(	VAMP7/M	18	-2.04515
BP	GO:00075	embryo im	0.00641	47/16305	0.000478	0.011202	ENSG0000(	CST3/MM	18	-1.95069
BP	GO:00193	hexose cat	0.00641	52/16305	0.001877	0.031485	ENSG0000(	PGAM1/EN	18	-1.50189
BP	GO:00506	regulation	0.006054	29/16305	6.23E-07	4.58E-05	ENSG0000(	AP2M1/AP	17	-4.33905
BP	GO:00027	MyD88-de	0.006054	36/16305	3.04E-05	0.001168	ENSG0000(	UBA52/CD	17	-2.93258
BP	GO:00450	actin nucle	0.006054	48/16305	0.001874	0.031485	ENSG0000(	ARPC3/AR	17	-1.50189
BP	GO:00458	negative r	0.006054	48/16305	0.001874	0.031485	ENSG0000(	TGFB1/SN	17	-1.50189

BP	GO:20004	regulation	0.006054	48/16305	0.001874	0.031485	ENSG0000	RHOA/ECI	17	-1.50189
BP	GO:00182	peptidyl-p	0.006054	49/16305	0.002416	0.036973	ENSG0000	P4HB/PPIE	17	-1.43212
BP	GO:00702	protein tri	0.006054	50/16305	0.003083	0.043735	ENSG0000	ITLN1/SIG	17	-1.35917
BP	GO:00726	interleukin	0.005698	28/16305	2.15E-06	0.000125	ENSG0000	TMSB4X/F	16	-3.90266
BP	GO:00716	regulation	0.005698	38/16305	0.000276	0.007536	ENSG0000	TGFB1/CM	16	-2.12288
BP	GO:00156	ferric iron	0.005698	39/16305	0.000394	0.009799	ENSG0000	STEAP3/AT	16	-2.00883
BP	GO:00330	regulation	0.005698	39/16305	0.000394	0.009799	ENSG0000	VAMP7/M	16	-2.00883
BP	GO:00725	trivalent ir	0.005698	39/16305	0.000394	0.009799	ENSG0000	STEAP3/AT	16	-2.00883
BP	GO:00458	negative r	0.005698	43/16305	0.001393	0.025468	ENSG0000	RPS19/NL	16	-1.59401
BP	GO:00327	positive re	0.005698	44/16305	0.001844	0.031279	ENSG0000	F2R/CD14	16	-1.50475
BP	GO:00901	regulation	0.005698	44/16305	0.001844	0.031279	ENSG0000	BMF/TRIA	16	-1.50475
BP	GO:00326	interleukin	0.005698	46/16305	0.003114	0.043957	ENSG0000	SASH3/PY	16	-1.35697
BP	GO:00457	respiratory	0.005342	29/16305	2.28E-05	0.000925	ENSG0000	RPS19/PG	15	-3.03387
BP	GO:00019	T cell med	0.005342	30/16305	3.83E-05	0.001446	ENSG0000	FADD/MIC	15	-2.83977
BP	GO:00335	transferrin	0.005342	36/16305	0.000489	0.011392	ENSG0000	STEAP3/AT	15	-1.94339
BP	GO:00430	T cell hom	0.005342	39/16305	0.001319	0.024273	ENSG0000	TGFB1/AK	15	-1.61487
BP	GO:00302	chondroiti	0.005342	41/16305	0.002365	0.036585	ENSG0000	CHPF2/CH	15	-1.4367
BP	GO:00325	protein ex	0.005342	41/16305	0.002365	0.036585	ENSG0000	ERLEC1/O	15	-1.4367
BP	GO:00066	unsaturate	0.005342	42/16305	0.003102	0.043892	ENSG0000	CD74/DEG	15	-1.35761
BP	GO:00702	regulation	0.004986	32/16305	0.000412	0.01014	ENSG0000	FADD/PRE	14	-1.99396
BP	GO:20004	regulation	0.004986	33/16305	0.000603	0.013285	ENSG0000	RHOA/ECI	14	-1.87663
BP	GO:00423	keratan su	0.004986	34/16305	0.000863	0.017407	ENSG0000	ACAN/GLI	14	-1.75927
BP	GO:20002	regulation	0.004986	35/16305	0.001213	0.022531	ENSG0000	TGFB1/AR	14	-1.64722
BP	GO:00508	regulation	0.004986	36/16305	0.001674	0.028651	ENSG0000	RAB29/KC	14	-1.54286
BP	GO:00190	virion asse	0.004986	37/16305	0.002272	0.03633	ENSG0000	UBA52/VP	14	-1.43974
BP	GO:00466	alpha-bet	0.00463	24/16305	4.28E-05	0.001596	ENSG0000	EBI3/CCR2	13	-2.79702
BP	GO:20004	regulation	0.00463	24/16305	4.28E-05	0.001596	ENSG0000	TMSB4X/F	13	-2.79702
BP	GO:00363	dendritic c	0.00463	27/16305	0.000206	0.005829	ENSG0000	GAS6/CAL	13	-2.2344
BP	GO:00903	phagosom	0.00463	27/16305	0.000206	0.005829	ENSG0000	ATP6V0D2	13	-2.2344
BP	GO:00309	retrograde	0.00463	28/16305	0.000324	0.008518	ENSG0000	ERLEC1/O	13	-2.06965
BP	GO:19035	endoplasn	0.00463	28/16305	0.000324	0.008518	ENSG0000	ERLEC1/O	13	-2.06965
BP	GO:00330	regulation	0.00463	29/16305	0.000495	0.011392	ENSG0000	VAMP7/IL	13	-1.94339
BP	GO:00425	superoxide	0.00463	29/16305	0.000495	0.011392	ENSG0000	TGFB1/AC	13	-1.94339
BP	GO:00433	regulation	0.00463	29/16305	0.000495	0.011392	ENSG0000	VAMP7/IL	13	-1.94339
BP	GO:00159	ATP hydro	0.00463	30/16305	0.000737	0.015504	ENSG0000	ATP6V0D2	13	-1.80955
BP	GO:00991	ATP hydro	0.00463	31/16305	0.001072	0.020454	ENSG0000	ATP6V0D2	13	-1.68922

BP	GO:00906	ATP hydrc	0.00463	32/16305	0.001523	0.026684	ENSG0000	ATP6V0D2	13	-1.57375
BP	GO:00159	energy co	0.00463	33/16305	0.002121	0.034688	ENSG0000	ATP6V0D2	13	-1.45982
BP	GO:00343	Arp2/3 co	0.00463	33/16305	0.002121	0.034688	ENSG0000	ARPC3/AR	13	-1.45982
BP	GO:00024	dendritic c	0.004274	23/16305	0.000137	0.00415	ENSG0000	GAS6/CAL	12	-2.38191
BP	GO:00070	vacuolar a	0.004274	23/16305	0.000137	0.00415	ENSG0000	PPT1/ATP	12	-2.38191
BP	GO:00550	response t	0.004274	23/16305	0.000137	0.00415	ENSG0000	CDH13/CD	12	-2.38191
BP	GO:00714	cellular res	0.004274	25/16305	0.000375	0.00952	ENSG0000	CDH13/CD	12	-2.02136
BP	GO:20004	positive re	0.004274	25/16305	0.000375	0.00952	ENSG0000	RHOA/FAI	12	-2.02136
BP	GO:00726	eosinophil	0.004274	29/16305	0.001904	0.031757	ENSG0000	CCL18/CD	12	-1.49817
BP	GO:00463	amino sug	0.003917	14/16305	8.47E-07	5.82E-05	ENSG0000	CHIT1/CTI	11	-4.23531
BP	GO:00466	regulation	0.003917	21/16305	0.00025	0.007018	ENSG0000	EBI3/CCR2	11	-2.15378
BP	GO:00315	membrane	0.003917	23/16305	0.000685	0.014668	ENSG0000	FLOT2/PPT	11	-1.83362
BP	GO:00104	proteason	0.003917	24/16305	0.001069	0.020454	ENSG0000	PSMB4/PS	11	-1.68922
BP	GO:00718	podosome	0.003917	24/16305	0.001069	0.020454	ENSG0000	RHOA/HC	11	-1.68922
BP	GO:00341	regulation	0.003917	25/16305	0.001615	0.027797	ENSG0000	CD9/LGAL	11	-1.55601
BP	GO:00603	cartilage c	0.003917	25/16305	0.001615	0.027797	ENSG0000	COMP/CA	11	-1.55601
BP	GO:00017	myeloid d	0.003917	26/16305	0.002368	0.036585	ENSG0000	TGFB1/SPI	11	-1.4367
BP	GO:00324	melanosor	0.003917	26/16305	0.002368	0.036585	ENSG0000	BLOC1S4/	11	-1.4367
BP	GO:00616	glycolytic	0.003917	26/16305	0.002368	0.036585	ENSG0000	PGAM1/EI	11	-1.4367
BP	GO:00616	glycolytic	0.003917	26/16305	0.002368	0.036585	ENSG0000	PGAM1/EI	11	-1.4367
BP	GO:00326	regulation	0.003917	27/16305	0.003381	0.046943	ENSG0000	SASH3/ND	11	-1.32843
BP	GO:00487	pigment g	0.003917	27/16305	0.003381	0.046943	ENSG0000	BLOC1S4/	11	-1.32843
BP	GO:00466	positive re	0.003561	17/16305	0.000135	0.00415	ENSG0000	EBI3/CCR2	10	-2.38191
BP	GO:00726	interferon	0.003561	17/16305	0.000135	0.00415	ENSG0000	TRIM27/G	10	-2.38191
BP	GO:00341	toll-like re	0.003561	19/16305	0.000459	0.010851	ENSG0000	FADD/UN	10	-1.96455
BP	GO:00513	kinetochoi	0.003561	21/16305	0.001255	0.023238	ENSG0000	NDC80/SN	10	-1.6338
BP	GO:00181	protein hy	0.003561	22/16305	0.001949	0.032136	ENSG0000	P4HB/CRT	10	-1.49301
BP	GO:00327	positive re	0.003561	22/16305	0.001949	0.032136	ENSG0000	SASH3/GA	10	-1.49301
BP	GO:00508	negative r	0.003561	22/16305	0.001949	0.032136	ENSG0000	DUSP3/TH	10	-1.49301
BP	GO:00019	regulation	0.003561	23/16305	0.002921	0.042144	ENSG0000	FADD/LILI	10	-1.37527
BP	GO:00026	regulation	0.003561	23/16305	0.002921	0.042144	ENSG0000	FADD/THY	10	-1.37527
BP	GO:20012	regulation	0.003561	23/16305	0.002921	0.042144	ENSG0000	MMP9/FA	10	-1.37527
BP	GO:00423	keratan su	0.003205	12/16305	1.75E-05	0.000738	ENSG0000	ACAN/GLI	9	-3.1321
BP	GO:00341	toll-like re	0.003205	13/16305	4.83E-05	0.001764	ENSG0000	IRAK1/CYI	9	-2.7534
BP	GO:00507	negative r	0.003205	14/16305	0.000114	0.003698	ENSG0000	TGFB1/SN	9	-2.43203
BP	GO:20004	positive re	0.003205	15/16305	0.000242	0.006821	ENSG0000	F2R/CD14	9	-2.16614

BP	GO:00021	store-ope	0.003205	17/16305	0.000845	0.017204	ENSG0000(	ORAI2/SA	9	-1.76437
BP	GO:00025	regulation	0.003205	17/16305	0.000845	0.017204	ENSG0000(	HLA-DOA	9	-1.76437
BP	GO:00193	leukotrien	0.003205	17/16305	0.000845	0.017204	ENSG0000(	MGST3/AL	9	-1.76437
BP	GO:00000	ribosomal	0.003205	18/16305	0.001432	0.025483	ENSG0000(	RPS19/RPS	9	-1.59376
BP	GO:00329	regulation	0.003205	18/16305	0.001432	0.025483	ENSG0000(	TGFB1/AC	9	-1.59376
BP	GO:00714	cellular res	0.003205	18/16305	0.001432	0.025483	ENSG0000(	CDH13/CD	9	-1.59376
BP	GO:00972	renal filtra	0.003205	18/16305	0.001432	0.025483	ENSG0000(	GAS6/F2R	9	-1.59376
BP	GO:00065	glycoprote	0.003205	19/16305	0.002308	0.03633	ENSG0000(	HPSE/CST	9	-1.43974
BP	GO:00069	pinocytosi	0.003205	19/16305	0.002308	0.03633	ENSG0000(	PPT1/NR1	9	-1.43974
BP	GO:00450	thymic T c	0.003205	19/16305	0.002308	0.03633	ENSG0000(	CD74/ATG	9	-1.43974
BP	GO:19016	glutathion	0.003205	19/16305	0.002308	0.03633	ENSG0000(	AKR1A1/M	9	-1.43974
BP	GO:19016	glutathion	0.003205	19/16305	0.002308	0.03633	ENSG0000(	AKR1A1/M	9	-1.43974
BP	GO:00324	regulation	0.003205	20/16305	0.003561	0.049209	ENSG0000(	BMF/GBA/	9	-1.30796
BP	GO:00026	positive re	0.002849	13/16305	0.000433	0.010405	ENSG0000(	FADD/THY	8	-1.98277
BP	GO:00195	peptidyl-p	0.002849	13/16305	0.000433	0.010405	ENSG0000(	P4HB/CRT	8	-1.98277
BP	GO:19041	regulation	0.002849	13/16305	0.000433	0.010405	ENSG0000(	ERLEC1/O	8	-1.98277
BP	GO:00019	negative r	0.002849	15/16305	0.001562	0.027197	ENSG0000(	HAVCR2/L	8	-1.56548
BP	GO:00901	chemokine	0.002849	16/16305	0.002654	0.039859	ENSG0000(	PYCARD/F	8	-1.39948
BP	GO:19010	glucosami	0.002493	10/16305	0.000329	0.008535	ENSG0000(	CHIT1/CTE	7	-2.06879
BP	GO:20011	regulation	0.002493	10/16305	0.000329	0.008535	ENSG0000(	TMEM176	7	-2.06879
BP	GO:00198	antigen pr	0.002493	11/16305	0.00077	0.016077	ENSG0000(	CD74/ATG	7	-1.7938
BP	GO:00433	negative T	0.002493	11/16305	0.00077	0.016077	ENSG0000(	CD74/ATG	7	-1.7938
BP	GO:00027	negative r	0.002493	12/16305	0.001574	0.027245	ENSG0000(	HAVCR2/L	7	-1.56471
BP	GO:00017	immunolo	0.002493	13/16305	0.002904	0.042144	ENSG0000(	NCK2/HAV	7	-1.37527
BP	GO:19055	positive re	0.002493	13/16305	0.002904	0.042144	ENSG0000(	CMKLR1/N	7	-1.37527
BP	GO:00017	membrane	0.002137	10/16305	0.002894	0.042144	ENSG0000(	FLOT2/FLC	6	-1.37527
BP	GO:00028	negative r	0.002137	10/16305	0.002894	0.042144	ENSG0000(	IL13RA2/C	6	-1.37527
BP	GO:00066	protein ret	0.002137	10/16305	0.002894	0.042144	ENSG0000(	GPAA1/OS	6	-1.37527
BP	GO:00320	regulation	0.002137	10/16305	0.002894	0.042144	ENSG0000(	AKT1/RPS	6	-1.37527
BP	GO:00450	negative t	0.002137	10/16305	0.002894	0.042144	ENSG0000(	CD74/ATG	6	-1.37527
BP	GO:19025	regulation	0.002137	10/16305	0.002894	0.042144	ENSG0000(	ITGB2/SYK	6	-1.37527
CC	GO:00059	focal adhe	0.049983	393/17241	6.13E-23	2.15E-20	ENSG0000(	TNS3/PFN	149	-19.6668
CC	GO:00059	cell-substr	0.049983	396/17241	1.47E-22	3.44E-20	ENSG0000(	TNS3/PFN	149	-19.463
CC	GO:00300	cell-substr	0.049983	401/17241	6.12E-22	1.08E-19	ENSG0000(	TNS3/PFN	149	-18.9681
CC	GO:00057	vacuolar n	0.046964	411/17241	7.16E-17	2.8E-15	ENSG0000(	HPSE/GNE	140	-14.5532
CC	GO:00444	endosoma	0.046964	489/17241	2.08E-10	4.88E-09	ENSG0000(	RAB31/VA	140	-8.31115

CC	GO:00985	side of me	0.045287	458/17241	5.06E-11	1.27E-09	ENSG0000(	P4HB/FCG	135	-8.896
CC	GO:00100	endosome	0.04428	448/17241	8.56E-11	2.08E-09	ENSG0000(	RAB31/VA	132	-8.68291
CC	GO:00310	extracellul	0.042603	466/17241	3.66E-08	6.44E-07	ENSG0000(	COMP/PC	127	-6.19137
CC	GO:00057	lysosomal	0.042268	353/17241	4.13E-17	1.93E-15	ENSG0000(	HPSE/GNE	126	-14.7136
CC	GO:00988	lytic vacuc	0.042268	353/17241	4.13E-17	1.93E-15	ENSG0000(	HPSE/GNE	126	-14.7136
CC	GO:00057	mitochonc	0.041932	500/17241	6.41E-06	7.77E-05	ENSG0000(	UCP2/PHE	125	-4.10934
CC	GO:00602	cytoplasm	0.040926	332/17241	9.69E-18	6.19E-16	ENSG0000(	CAP1/CTS	122	-15.2083
CC	GO:00319	vesicle lun	0.040926	333/17241	1.27E-17	6.89E-16	ENSG0000(	CAP1/CTS	122	-15.162
CC	GO:00347	secretory (	0.038913	315/17241	5.19E-17	2.28E-15	ENSG0000(	CAP1/CTS	116	-14.6424
CC	GO:00306	secretory (	0.037236	297/17241	7.14E-17	2.8E-15	ENSG0000(	RAB31/CD	111	-14.5532
CC	GO:00057	endoplasn	0.036229	291/17241	3.33E-16	1.23E-14	ENSG0000(	P4HB/QSC	108	-13.9095
CC	GO:00156	actin cytos	0.035894	485/17241	0.00363	0.021998	ENSG0000(	CAP1/TMS	107	-1.65762
CC	GO:00058	ribosome	0.033546	237/17241	1.33E-19	1.04E-17	ENSG0000(	RPL18/RPI	100	-16.9846
CC	GO:00055	proteinace	0.032204	366/17241	9.61E-06	0.000111	ENSG0000(	COMP/PC	96	-3.95547
CC	GO:00444	cytosolic p	0.031533	248/17241	6.18E-15	2.07E-13	ENSG0000(	RPL18/RPI	94	-12.6843
CC	GO:00443	ribosomal	0.030862	188/17241	1.24E-23	8.72E-21	ENSG0000(	RPL18/RPI	92	-20.0594
CC	GO:00985	membrane	0.030191	315/17241	3.88E-07	5.93E-06	ENSG0000(	CTSD/HPS	90	-5.22728
CC	GO:00301	transport '	0.030191	371/17241	0.000363	0.003355	ENSG0000(	TMED9/PF	90	-2.47433
CC	GO:00301	endocytic	0.02952	286/17241	1.29E-08	2.59E-07	ENSG0000(	ITGB5/RAE	88	-6.58658
CC	GO:00451	membrane	0.02952	303/17241	2.38E-07	3.8E-06	ENSG0000(	CTSD/HPS	88	-5.41978
CC	GO:00988	membrane	0.02952	304/17241	2.79E-07	4.36E-06	ENSG0000(	CTSD/HPS	88	-5.36053
CC	GO:01010	ficolin-1-r	0.028849	182/17241	6.7E-21	7.85E-19	ENSG0000(	CTSD/CTS	86	-18.1052
CC	GO:00057	vacuolar lu	0.027843	169/17241	1.4E-21	1.96E-19	ENSG0000(	CAP1/CTS	83	-18.7067
CC	GO:00301	coated ves	0.026501	270/17241	6.98E-07	1E-05	ENSG0000(	TMED9/CT	79	-4.9992
CC	GO:00057	early endo	0.026166	332/17241	0.002226	0.014628	ENSG0000(	RAB31/RA	78	-1.83481
CC	GO:00098	external si	0.02583	240/17241	1.41E-08	2.76E-07	ENSG0000(	P4HB/FCG	77	-6.55927
CC	GO:00057	primary ly:	0.025159	152/17241	8.74E-20	7.68E-18	ENSG0000(	CAP1/DD(	75	-17.1148
CC	GO:00425	azurophil (	0.025159	152/17241	8.74E-20	7.68E-18	ENSG0000(	CAP1/DD(	75	-17.1148
CC	GO:00987	mitochonc	0.025159	262/17241	3.25E-06	4.15E-05	ENSG0000(	GADD45G	75	-4.382
CC	GO:00058	spindle	0.024824	321/17241	0.004689	0.027471	ENSG0000(	CAPG/MZ	74	-1.56113
CC	GO:00708	tertiary gr	0.022811	161/17241	8.43E-14	2.69E-12	ENSG0000(	CTSD/QSC	68	-11.5699
CC	GO:00226	cytosolic r	0.020798	118/17241	2.42E-18	1.7E-16	ENSG0000(	RPL18/RPI	62	-15.7686
CC	GO:00057	late endos	0.020798	239/17241	0.000465	0.003989	ENSG0000(	SDF4/VAN	62	-2.39914
CC	GO:19048	ficolin-1-r	0.020127	124/17241	1.32E-15	4.65E-14	ENSG0000(	CTSD/CTS	60	-13.3321
CC	GO:00425	specific gr	0.020127	158/17241	4.39E-10	9.96E-09	ENSG0000(	CTSD/HPS	60	-8.00164
CC	GO:00159	large ribos	0.01845	118/17241	1.46E-13	4.47E-12	ENSG0000(	RPL18/RPI	55	-11.3496

CC	GO:00306	endocytic	0.018115	157/17241	1.61E-07	2.63E-06	ENSG0000(	RAB31/VA	54	-5.57926
CC	GO:00432	lysosomal	0.017779	94/17241	1.15E-17	6.74E-16	ENSG0000(	CTSD/HPS	53	-15.1714
CC	GO:00453	phagocyti	0.017779	123/17241	1.8E-11	5.05E-10	ENSG0000(	ITGB5/RAI	53	-9.2963
CC	GO:00444	mitochonc	0.017779	218/17241	0.005078	0.028335	ENSG0000(	IMMT/NO	53	-1.54768
CC	GO:00300	lamellipoc	0.017444	188/17241	0.000252	0.002424	ENSG0000(	FLOT2/FAI	52	-2.61551
CC	GO:00058	trans-Golg	0.017444	199/17241	0.00107	0.007757	ENSG0000(	RAB31/VA	52	-2.11033
CC	GO:00057	Golgi-ass(	0.017108	168/17241	2.09E-05	0.000237	ENSG0000(	TMED9/CT	51	-3.62562
CC	GO:00988	plasma me	0.016773	180/17241	0.000293	0.002786	ENSG0000(	ITGB5/CHI	50	-2.55508
CC	GO:00306	coated ves	0.016102	165/17241	0.000116	0.001221	ENSG0000(	VAMP7/H	48	-2.91314
CC	GO:00312	intrinsic cc	0.015431	150/17241	3.92E-05	0.000438	ENSG0000(	SPCS1/PIG	46	-3.35878
CC	GO:00301	clathrin-cc	0.015431	175/17241	0.001765	0.012049	ENSG0000(	TMED9/VA	46	-1.91903
CC	GO:00301	integral cc	0.015096	146/17241	4.13E-05	0.000453	ENSG0000(	SPCS1/PIG	45	-3.34343
CC	GO:00355	azurophil	0.014425	89/17241	1.47E-11	4.32E-10	ENSG0000(	CAP1/ADA	43	-9.36486
CC	GO:00304	midbody	0.014425	166/17241	0.003279	0.020219	ENSG0000(	CAPG/RH(	43	-1.69425
CC	GO:00424	melanosoı	0.013754	105/17241	9.94E-08	1.66E-06	ENSG0000(	CTSD/P4H	41	-5.77909
CC	GO:00487	pigment g	0.013754	105/17241	9.94E-08	1.66E-06	ENSG0000(	CTSD/P4H	41	-5.77909
CC	GO:00355	specific gr	0.012747	89/17241	1.71E-08	3.16E-07	ENSG0000(	ALDH3B1/	38	-6.50076
CC	GO:00988	inner mito	0.012747	141/17241	0.002645	0.016907	ENSG0000(	IMMT/CO)	38	-1.77194
CC	GO:00159	small ribos	0.012412	71/17241	2.25E-11	5.98E-10	ENSG0000(	RPS19/RPS	37	-9.22294
CC	GO:19902	oxidoredu	0.012076	111/17241	7.33E-05	0.000793	ENSG0000(	P4HB/RRN	36	-3.10085
CC	GO:00319	late endos	0.012076	124/17241	0.000815	0.006297	ENSG0000(	VAMP7/YI	36	-2.20084
CC	GO:00007	kinetochoı	0.012076	132/17241	0.002715	0.017196	ENSG0000(	SUMO3/M	36	-1.76458
CC	GO:00226	cytosolic l	0.011741	65/17241	2.3E-11	5.98E-10	ENSG0000(	RPL18/RPI	35	-9.22294
CC	GO:00057	endoplasn	0.011741	120/17241	0.000873	0.006596	ENSG0000(	TMED9/P4	35	-2.1807
CC	GO:00007	condense(	0.011406	116/17241	0.000933	0.006905	ENSG0000(	CBX3/MEA	34	-2.16081
CC	GO:00444	extracellul	0.011406	118/17241	0.001294	0.009004	ENSG0000(	MFAP5/CS	34	-2.04559
CC	GO:00306	phagocyti	0.01107	71/17241	1.16E-08	2.39E-07	ENSG0000(	RAB31/VA	33	-6.62105
CC	GO:00003	organellar	0.01107	85/17241	1.99E-06	2.64E-05	ENSG0000(	GADD45G	33	-4.57839
CC	GO:00057	mitochonc	0.01107	85/17241	1.99E-06	2.64E-05	ENSG0000(	GADD45G	33	-4.57839
CC	GO:00301	COPII-coa	0.010735	86/17241	8.12E-06	9.52E-05	ENSG0000(	TMED9/CT	32	-4.0214
CC	GO:00704	respiratory	0.010399	97/17241	0.000302	0.002833	ENSG0000(	OXA1L/CC	31	-2.54772
CC	GO:00007	condense(	0.010399	103/17241	0.00095	0.006957	ENSG0000(	MEAF6/NI	31	-2.15761
CC	GO:00306	Golgi-ass(	0.010399	107/17241	0.001877	0.012568	ENSG0000(	HLA-DQB	31	-1.90073
CC	GO:00310	platelet al	0.010064	91/17241	0.000204	0.001996	ENSG0000(	TMSB4X/C	30	-2.69991
CC	GO:00708	tertiary gr	0.009728	71/17241	2.46E-06	3.21E-05	ENSG0000(	ATP6AP2/	29	-4.49391
CC	GO:00057	mitochonc	0.009728	91/17241	0.000491	0.00416	ENSG0000(	OXA1L/CC	29	-2.38087

CC	GO:00306 clathrin-cc	0.009728	100/17241	0.002543	0.016399	ENSG0000(	VAMP7/H	29	-1.78519
CC	GO:00058 actin filam	0.009728	102/17241	0.003499	0.021392	ENSG0000(	TMSB4X/T	29	-1.66976
CC	GO:00355 azurophil	0.009393	57/17241	3.28E-08	5.92E-07	ENSG0000(	DDOST/V/	28	-6.22776
CC	GO:19053 peptidase	0.009393	91/17241	0.001126	0.008077	ENSG0000(	SPCS1/SP(	28	-2.09275
CC	GO:00226 cytosolic s	0.008722	46/17241	2.12E-09	4.66E-08	ENSG0000(	RPS19/RPS	26	-7.33129
CC	GO:01010 ficolin-1-r	0.008722	58/17241	9.75E-07	1.37E-05	ENSG0000(	RHOA/ATI	26	-4.86308
CC	GO:00988 respiratory	0.008722	84/17241	0.001512	0.010419	ENSG0000(	COX5A/NI	26	-1.98217
CC	GO:19047 tertiary gra	0.008051	56/17241	6.64E-06	7.91E-05	ENSG0000(	CTSD/QS(	24	-4.10178
CC	GO:00331 endoplasn	0.007716	66/17241	0.000447	0.003881	ENSG0000(	TMED9/CT	23	-2.4111
CC	GO:19053 endopepti	0.007716	67/17241	0.000571	0.004777	ENSG0000(	PSMC2/PS	23	-2.32082
CC	GO:00005 proteasom	0.00738	66/17241	0.001165	0.008274	ENSG0000(	PSMC2/PS	22	-2.0823
CC	GO:00550 recycling e	0.00738	69/17241	0.002225	0.014628	ENSG0000(	PDIA3/VA	22	-1.83481
CC	GO:00125 ER to Golg	0.006709	58/17241	0.001197	0.008412	ENSG0000(	HLA-DQB	20	-2.07509
CC	GO:00355 specific gr	0.006709	62/17241	0.002969	0.018471	ENSG0000(	CTSD/HPS	20	-1.73351
CC	GO:00003 organellar	0.006374	52/17241	0.000704	0.005626	ENSG0000(	MRPL34/N	19	-2.24983
CC	GO:00057 mitochonc	0.006374	52/17241	0.000704	0.005626	ENSG0000(	MRPL34/N	19	-2.24983
CC	GO:00164 proton-tra	0.006374	52/17241	0.000704	0.005626	ENSG0000(	ATP6V0D2	19	-2.24983
CC	GO:00017 immunolo	0.006038	33/17241	1.33E-06	1.83E-05	ENSG0000(	STOML2/(	18	-4.73713
CC	GO:00986 protein co	0.006038	35/17241	4.09E-06	5.13E-05	ENSG0000(	ITGB5/ITG	18	-4.28983
CC	GO:00083 integrin cc	0.005703	32/17241	4.25E-06	5.24E-05	ENSG0000(	ITGB5/ITG	17	-4.28028
CC	GO:00321 cleavage f	0.005703	54/17241	0.007748	0.041913	ENSG0000(	RHOA/ITG	17	-1.37766
CC	GO:00426 MHC prote	0.005367	22/17241	1.6E-08	3.03E-07	ENSG0000(	HLA-DQB	16	-6.51789
CC	GO:00715 integral cc	0.005367	26/17241	5.63E-07	8.24E-06	ENSG0000(	HLA-DQB	16	-5.08388
CC	GO:00985 lumenal si	0.005367	26/17241	5.63E-07	8.24E-06	ENSG0000(	HLA-DQB	16	-5.08388
CC	GO:00421 T cell rece	0.005032	19/17241	6.87E-09	1.46E-07	ENSG0000(	STOML2/S	15	-6.83432
CC	GO:00306 clathrin-cc	0.005032	40/17241	0.001855	0.012539	ENSG0000(	HLA-DQB	15	-1.90174
CC	GO:00427 polysomal	0.004361	30/17241	0.000767	0.006061	ENSG0000(	RPL18/RPI	13	-2.21745
CC	GO:00331 proton-tra	0.004025	26/17241	0.000611	0.005053	ENSG0000(	ATP6V0D2	12	-2.29647
CC	GO:00700 cytochrom	0.004025	31/17241	0.003828	0.023003	ENSG0000(	COX5A/C1	12	-1.63821
CC	GO:00021 podosome	0.004025	34/17241	0.008984	0.047488	ENSG0000(	ACTR2/FEI	12	-1.32341
CC	GO:00058 proteasom	0.00369	22/17241	0.000439	0.003881	ENSG0000(	PSMB4/PS	11	-2.4111
CC	GO:00003 organellar	0.00369	28/17241	0.004866	0.027812	ENSG0000(	MRPS12/N	11	-1.55576
CC	GO:00057 mitochonc	0.00369	28/17241	0.004866	0.027812	ENSG0000(	MRPS12/N	11	-1.55576
CC	GO:00443 endoplasn	0.00369	28/17241	0.004866	0.027812	ENSG0000(	ERLEC1/U	11	-1.55576
CC	GO:00310 BLOC com	0.003355	20/17241	0.000803	0.00627	ENSG0000(	BLOC1S4/	10	-2.20271
CC	GO:00301 COPI-coat	0.003355	25/17241	0.006174	0.034176	ENSG0000(	TMED3/C(	10	-1.46628

CC	GO:00426	MHC class	0.003019	14/17241	0.000118	0.001222	ENSG0000(	HLA-DQB	9	-2.91305
CC	GO:00310	platelet alı	0.003019	17/17241	0.000871	0.006596	ENSG0000(	CD109/SP	9	-2.1807
CC	GO:00360	endolysos	0.003019	19/17241	0.002376	0.015465	ENSG0000(	CTSB/AP2	9	-1.81065
CC	GO:00007	condense(	0.003019	22/17241	0.00781	0.041913	ENSG0000(	NDC80/BU	9	-1.37766
CC	GO:00309	retromer (	0.003019	22/17241	0.00781	0.041913	ENSG0000(	SNX3/VPS	9	-1.37766
CC	GO:00312	lamellipod	0.003019	22/17241	0.00781	0.041913	ENSG0000(	FAP/PLXN	9	-1.37766
CC	GO:00197	proteasom	0.002684	11/17241	7.95E-05	0.000846	ENSG0000(	PSMB4/PS	8	-3.07245
CC	GO:00007	condense(	0.002684	12/17241	0.000202	0.001996	ENSG0000(	NDC80/BU	8	-2.69991
CC	GO:00082	oligosacch	0.002684	12/17241	0.000202	0.001996	ENSG0000(	DDOST/D/	8	-2.69991
CC	GO:00430	NADPH ox	0.002684	12/17241	0.000202	0.001996	ENSG0000(	NOX4/CYI	8	-2.69991
CC	GO:00009	condense(	0.002684	13/17241	0.000446	0.003881	ENSG0000(	NDC80/BU	8	-2.4111
CC	GO:00019	uropod	0.002684	13/17241	0.000446	0.003881	ENSG0000(	FLOT2/SC	8	-2.4111
CC	GO:00312	cell trailing	0.002684	13/17241	0.000446	0.003881	ENSG0000(	FLOT2/SC	8	-2.4111
CC	GO:00057	secondary	0.002684	14/17241	0.000882	0.006597	ENSG0000(	FTL/FTH1/	8	-2.18066
CC	GO:00058	eukaryotic	0.002684	17/17241	0.004374	0.025838	ENSG0000(	EIF3G/EIF3	8	-1.58775
CC	GO:00331	proton-tra	0.002684	17/17241	0.004374	0.025838	ENSG0000(	ATP6AP1/	8	-1.58775
CC	GO:00057	mitochonc	0.002348	14/17241	0.005067	0.028335	ENSG0000(	UQCRQ/U	7	-1.54768
CC	GO:00452	respiratory	0.002348	14/17241	0.005067	0.028335	ENSG0000(	UQCRQ/U	7	-1.54768
CC	GO:00310	BLOC-1 co	0.002348	15/17241	0.008096	0.043116	ENSG0000(	BLOC1S4/	7	-1.36536
CC	GO:00331	proton-tra	0.002013	10/17241	0.002956	0.018471	ENSG0000(	ATP6V0D2	6	-1.73351
MF	GO:00508	cell adhesi	0.043987	487/16312	2.54E-06	0.000408	ENSG0000(	PFN1/P4H	124	-3.38954
MF	GO:00037	structural	0.029798	156/16312	2.35E-25	2.64E-22	ENSG0000(	RPL18/RPI	84	-21.5783
MF	GO:00167	transferase	0.027315	264/16312	1.07E-06	0.000215	ENSG0000(	CHPF2/DD	77	-3.66755
MF	GO:00302	carbohydr	0.025186	260/16312	3.25E-05	0.002434	ENSG0000(	GALNT5/IT	71	-2.6136
MF	GO:00055	glycosami	0.020929	202/16312	1.77E-05	0.001658	ENSG0000(	COMP/PC	59	-2.78047
MF	GO:00167	transferase	0.019156	192/16312	0.000119	0.006351	ENSG0000(	CHPF2/DD	54	-2.19719
MF	GO:00168	isomerase	0.015254	142/16312	9.39E-05	0.00528	ENSG0000(	P4HB/QSC	43	-2.27738
MF	GO:00082	heparin bi	0.014544	151/16312	0.001563	0.040889	ENSG0000(	COMP/PC	41	-1.38839
MF	GO:00051	integrin bi	0.014189	117/16312	7.21E-06	0.000738	ENSG0000(	P4HB/FAP	40	-3.13211
MF	GO:00090	electron tr	0.01348	109/16312	7.2E-06	0.000738	ENSG0000(	SH3BGRL3	38	-3.13211
MF	GO:00167	hydrolase	0.01348	123/16312	0.000147	0.006635	ENSG0000(	HPSE/MP(	38	-2.17816
MF	GO:00150	proton tra	0.01277	127/16312	0.001294	0.035512	ENSG0000(	SLC15A3/	36	-1.44963
MF	GO:00199	cytokine b	0.012061	102/16312	6.12E-05	0.003827	ENSG0000(	CD109/LT	34	-2.41715
MF	GO:00045	hydrolase	0.011352	97/16312	0.000124	0.006351	ENSG0000(	HPSE/PSA	32	-2.19719
MF	GO:00055	collagen b	0.009933	63/16312	4.62E-07	0.000215	ENSG0000(	COMP/PP	28	-3.66755
MF	GO:00166	oxidoredu	0.009223	61/16312	3.1E-06	0.000436	ENSG0000(	P4HB/QSC	26	-3.36092

MF	GO:00198	rRNA binc	0.008868	58/16312	3.74E-06	0.000467	ENSG0000	RPS9/NOF	25	-3.33062
MF	GO:00052	extracellul	0.008514	74/16312	0.001093	0.031933	ENSG0000	COMP/MF	24	-1.49576
MF	GO:00168	intramolec	0.008159	47/16312	5.94E-07	0.000215	ENSG0000	P4HB/QSC	23	-3.66755
MF	GO:00480	monosacc	0.008159	70/16312	0.001127	0.031933	ENSG0000	P3H1/LMA	23	-1.49576
MF	GO:00150	disulfide o	0.00674	43/16312	3.54E-05	0.002492	ENSG0000	P4HB/SH3	19	-2.60345
MF	GO:00166	oxidoredu	0.006385	46/16312	0.000366	0.013741	ENSG0000	MGST3/PF	18	-1.86198
MF	GO:00083	acetylgala	0.006385	47/16312	0.000499	0.01649	ENSG0000	CHPF2/CH	18	-1.78278
MF	GO:00038	antigen bi	0.006385	52/16312	0.001955	0.046785	ENSG0000	TGFB1/HL	18	-1.32989
MF	GO:00046	peroxidase	0.006031	42/16312	0.000331	0.012835	ENSG0000	MGST3/PF	17	-1.89162
MF	GO:00328	tumor nec	0.006031	46/16312	0.001135	0.031933	ENSG0000	TNFSF12/T	17	-1.49576
MF	GO:00447	ATPase ac	0.005676	34/16312	5.72E-05	0.003787	ENSG0000	ATP6V0D2	16	-2.42174
MF	GO:00422	MHC prot	0.005321	29/16312	2.38E-05	0.002047	ENSG0000	CD74/BCA	15	-2.68895
MF	GO:00051	tumor nec	0.004966	31/16312	0.000285	0.011467	ENSG0000	TNFSF12/T	14	-1.94055
MF	GO:19015	carbohydr	0.004966	36/16312	0.001733	0.042384	ENSG0000	SLC29A1/S	14	-1.3728
MF	GO:00037	protein dis	0.004612	19/16312	1.15E-06	0.000215	ENSG0000	P4HB/QSC	13	-3.66755
MF	GO:00168	intramolec	0.004612	19/16312	1.15E-06	0.000215	ENSG0000	P4HB/QSC	13	-3.66755
MF	GO:00364	proton-ex	0.004612	29/16312	0.000513	0.01649	ENSG0000	ATP6V0D2	13	-1.78278
MF	GO:00198	immunogl	0.003902	19/16312	7.78E-05	0.004604	ENSG0000	FCGR3A/F	11	-2.33683
MF	GO:00042	threonine-	0.003902	20/16312	0.000146	0.006635	ENSG0000	PSMB4/PS	11	-2.17816
MF	GO:00700	threonine-	0.003902	20/16312	0.000146	0.006635	ENSG0000	PSMB4/PS	11	-2.17816
MF	GO:00469	proton-tra	0.003902	21/16312	0.000258	0.011031	ENSG0000	ATP6V0D2	11	-1.95738
MF	GO:00199	chemokine	0.003902	25/16312	0.001663	0.041576	ENSG0000	ACKR4/A2	11	-1.38116
MF	GO:00046	glutathion	0.003547	18/16312	0.000265	0.011031	ENSG0000	MGST3/GI	10	-1.95738
MF	GO:00055	hyaluronic	0.003547	20/16312	0.000799	0.0243	ENSG0000	HAPLN1/A	10	-1.6144
MF	GO:00084	sulfuric es	0.003193	16/16312	0.000482	0.016423	ENSG0000	SGSH/GNS	9	-1.78453
MF	GO:00506	oxidoredu	0.003193	16/16312	0.000482	0.016423	ENSG0000	NOX4/CYI	9	-1.78453
MF	GO:00422	MHC class	0.003193	18/16312	0.001469	0.03936	ENSG0000	BCAP31/P	9	-1.40494
MF	GO:00045	oligosacch	0.002838	10/16312	2.55E-05	0.002047	ENSG0000	DDOST/D	8	-2.68895
MF	GO:00150	peptide di	0.002838	13/16312	0.000444	0.016108	ENSG0000	P4HB/PDI	8	-1.79295
MF	GO:00161	superoxide	0.002483	11/16312	0.000787	0.0243	ENSG0000	NOX4/CYI	7	-1.6144
MF	GO:00315	peptidyl-p	0.002483	12/16312	0.001607	0.0411	ENSG0000	P4HB/P3H	7	-1.38616
