# Supplementary material for: Single-cell atlas reveals different immune environments between stable and vulnerable atherosclerotic plaques
Source: Front Immunol. 2023 Jan 18;13:1085468. doi: 10.3389/fimmu.2022.1085468 (PMC9889979; doi:10.3389/fimmu.2022.1085468)
Supplement: Supplementary file 2 [file DataSheet_2.pdf]

KEGGID	Descriptio	GeneRatic	BgRatio	pvalue	padj	geneID	geneNam	keggID	Count	LogFDR
hsa05034	Alcoholism	75/1221	167/6777	4.08E-16	1.28E-13	ENSG0000	HIST2H2B	hsa:44068	75	-12.8933
hsa05322	Systemic l	54/1221	104/6777	3.05E-15	4.77E-13	ENSG0000	HIST2H2B	hsa:44068	54	-12.3218
hsa05033	Nicotine a	28/1221	40/6777	6.61E-13	6.9E-11	ENSG0000	GRIN2A/G	hsa:2903/	28	-10.1612
hsa04080	Neuroacti	95/1221	264/6777	1.06E-12	8.26E-11	ENSG0000	GRIN2A/G	hsa:2903/	95	-10.0832
hsa04727	GABAergi	42/1221	86/6777	5.15E-11	3.22E-09	ENSG0000	GABRA2/S	hsa:2555/	42	-8.49197
hsa04742	Taste tran	33/1221	64/6777	1.02E-09	5.33E-08	ENSG0000	ENTPD2/G	hsa:954/hs	33	-7.2731
hsa04724	Glutamate	44/1221	114/6777	1.48E-07	6.62E-06	ENSG0000	GRIN2A/S	hsa:2903/	44	-5.17897
hsa04024	cAMP sigr	62/1221	191/6777	7.55E-07	2.95E-05	ENSG0000	GRIN2A/A	hsa:2903/	62	-4.52962
hsa04713	Circadian	35/1221	95/6777	9.44E-06	0.000328	ENSG0000	GRIN2A/C	hsa:2903/	35	-3.48376
hsa05032	Morphine	33/1221	89/6777	1.45E-05	0.000428	ENSG0000	GABRA2/C	hsa:2555/	33	-3.36901
hsa04976	Bile secret	27/1221	67/6777	1.5E-05	0.000428	ENSG0000	ATP1B2/A	hsa:482/hs	27	-3.36901
hsa04911	Insulin sec	29/1221	80/6777	7.63E-05	0.00199	ENSG0000	ATP1B2/C	hsa:482/hs	29	-2.70119
hsa04971	Gastric aci	27/1221	73/6777	8.99E-05	0.002166	ENSG0000	ATP1B2/C	hsa:482/hs	27	-2.66441
hsa04020	Calcium si	53/1221	182/6777	0.000136	0.003043	ENSG0000	GRIN2A/C	hsa:2903/	53	-2.51663
hsa04726	Serotoner	36/1221	112/6777	0.000196	0.00394	ENSG0000	CACNA1A	hsa:773/hs	36	-2.40453
hsa04964	Proximal t	12/1221	23/6777	0.000211	0.00394	ENSG0000	ATP1B2/A	hsa:482/hs	12	-2.40453
hsa04723	Retrograd	44/1221	146/6777	0.000214	0.00394	ENSG0000	GABRA2/C	hsa:2555/	44	-2.40453
hsa04977	Vitamin di	11/1221	23/6777	0.001004	0.017466	ENSG0000	SLC19A3/S	hsa:80704	11	-1.75781
hsa00910	Nitrogen r	9/1221	17/6777	0.001176	0.019366	ENSG0000	CA4/CPS1	hsa:762/hs	9	-1.71296
hsa04725	Cholinergi	32/1221	107/6777	0.001717	0.026864	ENSG0000	CAMK2A/C	hsa:815/hs	32	-1.57082
hsa04721	Synaptic v	19/1221	56/6777	0.003099	0.044197	ENSG0000	SYT1/CPLX	hsa:6857/	19	-1.35461
hsa04974	Protein dig	26/1221	85/6777	0.003185	0.044197	ENSG0000	ATP1B2/C	hsa:482/hs	26	-1.35461
hsa04390	Hippo sigr	41/1221	151/6777	0.003248	0.044197	ENSG0000	CTNNA2/S	hsa:1496/	41	-1.35461
hsa03010	Ribosome	75/1318	128/6777	6.4E-23	2E-20	ENSG0000	RPL18/RPI	hsa:6141/	75	19.69854
hsa04142	Lysosome	65/1318	121/6777	2.51E-17	3.93E-15	ENSG0000	CTSD/CTS	hsa:1509/	65	14.40566
hsa04145	Phagosom	51/1318	125/6777	2.39E-08	2.5E-06	ENSG0000	COMP/ITG	hsa:1311/	51	5.602736
hsa05110	Vibrio cho	25/1318	47/6777	2.37E-07	1.85E-05	ENSG0000	GNAS/ATF	hsa:2778/	25	4.73264
hsa04650	Natural kil	19/1318	34/6777	2.53E-06	0.000158	ENSG0000	FCGR3A/IT	hsa:2214/	19	3.800532
hsa00520	Amino sug	21/1318	46/6777	4.63E-05	0.00226	ENSG0000	CHIT1/CYE	hsa:1118/	21	2.645798
hsa04670	Leukocyte	38/1318	106/6777	5.06E-05	0.00226	ENSG0000	MMP2/RH	hsa:4313/	38	2.645798
hsa05152	Tuberculo	50/1318	154/6777	7.4E-05	0.002896	ENSG0000	CTSD/FCG	hsa:1509/	50	2.538127
hsa04668	TNF signa	18/1318	38/6777	8.88E-05	0.003087	ENSG0000	AKT1/MM	hsa:207/hs	15	2.510464
hsa00531	Glycosami	11/1318	18/6777	0.000119	0.00373	ENSG0000	HPSE/SGS	hsa:10855	11	2.428332
hsa04666	Fc gamma	30/1318	81/6777	0.000157	0.004333	ENSG0000	FCGR3A/A	hsa:2214/	24	2.363231
hsa04611	Platelet ac	40/1318	119/6777	0.000166	0.004333	ENSG0000	GNAS/RH	hsa:2778/	40	2.363231

hsa04141	Protein pr	49/1318	156/6777	0.000217	0.005215	ENSG0000	P4HB/DD(	hsa:5034/	49	2.282758
hsa05323	Rheumato	26/1318	69/6777	0.000308	0.00689	ENSG0000	TGFB1/AT	hsa:7040/	26	2.161785
hsa04658	Th1 and T	41/1318	128/6777	0.000441	0.009204	ENSG0000	IL2RA/GA	hsa:3559/	18	2.036031
hsa00511	Other glyc	9/1318	15/6777	0.000623	0.012181	ENSG0000	FUCA1/M	hsa:2517/	9	1.914323
hsa04662	B cell rece	58/1318	204/6777	0.001065	0.019609	ENSG0000	AKT1/CD8	hsa:207/hs	19	1.707544
hsa05140	Leishmani	21/1318	58/6777	0.002046	0.03376	ENSG0000	FCGR3A/T	hsa:2214/	21	1.471599
hsa04660	T cell rece	10/1318	20/6777	0.002049	0.03376	ENSG0000	CHPF2/CH	hsa:54480	24	1.471599
hsa04380	Osteoclast	34/1318	110/6777	0.002575	0.040306	ENSG0000	FCGR3A/T	hsa:2214/	34	1.394634
