# Supplementary figures and images for: Single-cell atlas reveals different immune environments between stable and vulnerable atherosclerotic plaques
Source: Front Immunol. 2023 Jan 18;13:1085468. doi: 10.3389/fimmu.2022.1085468 (PMC9889979; doi:10.3389/fimmu.2022.1085468)

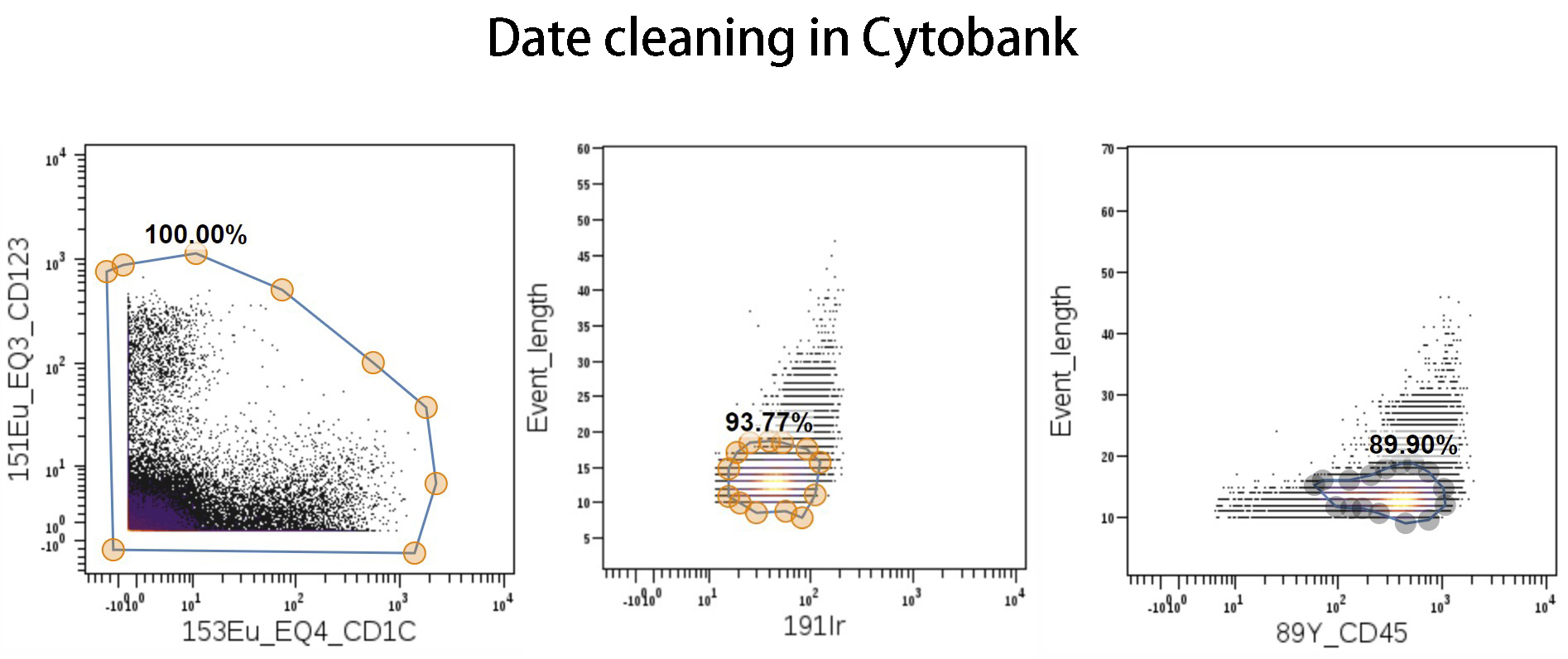

Supplement: Supplementary file 4 [file Image_1.jpeg]

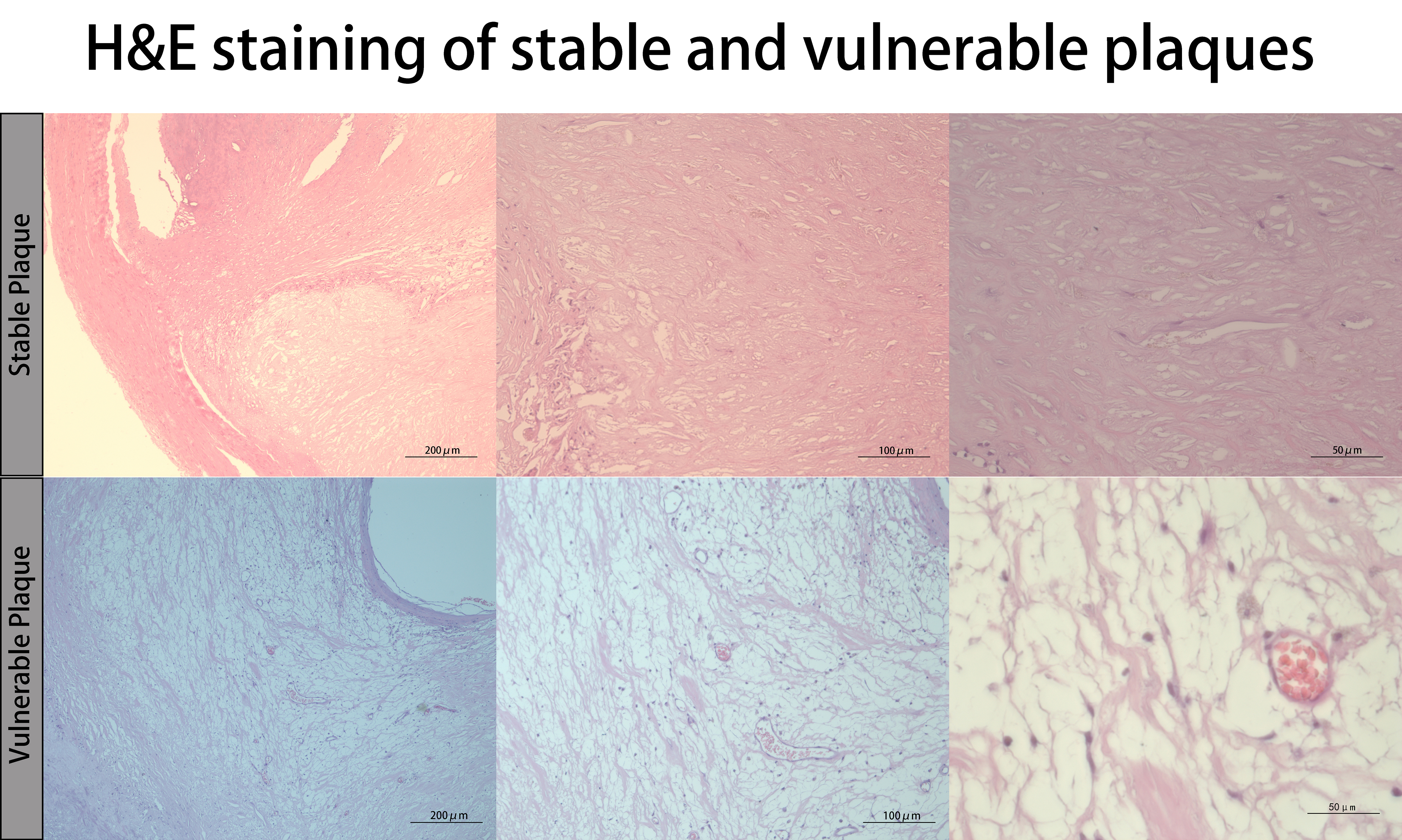

Supplement: Supplementary file 5 [file Image_2.jpeg]

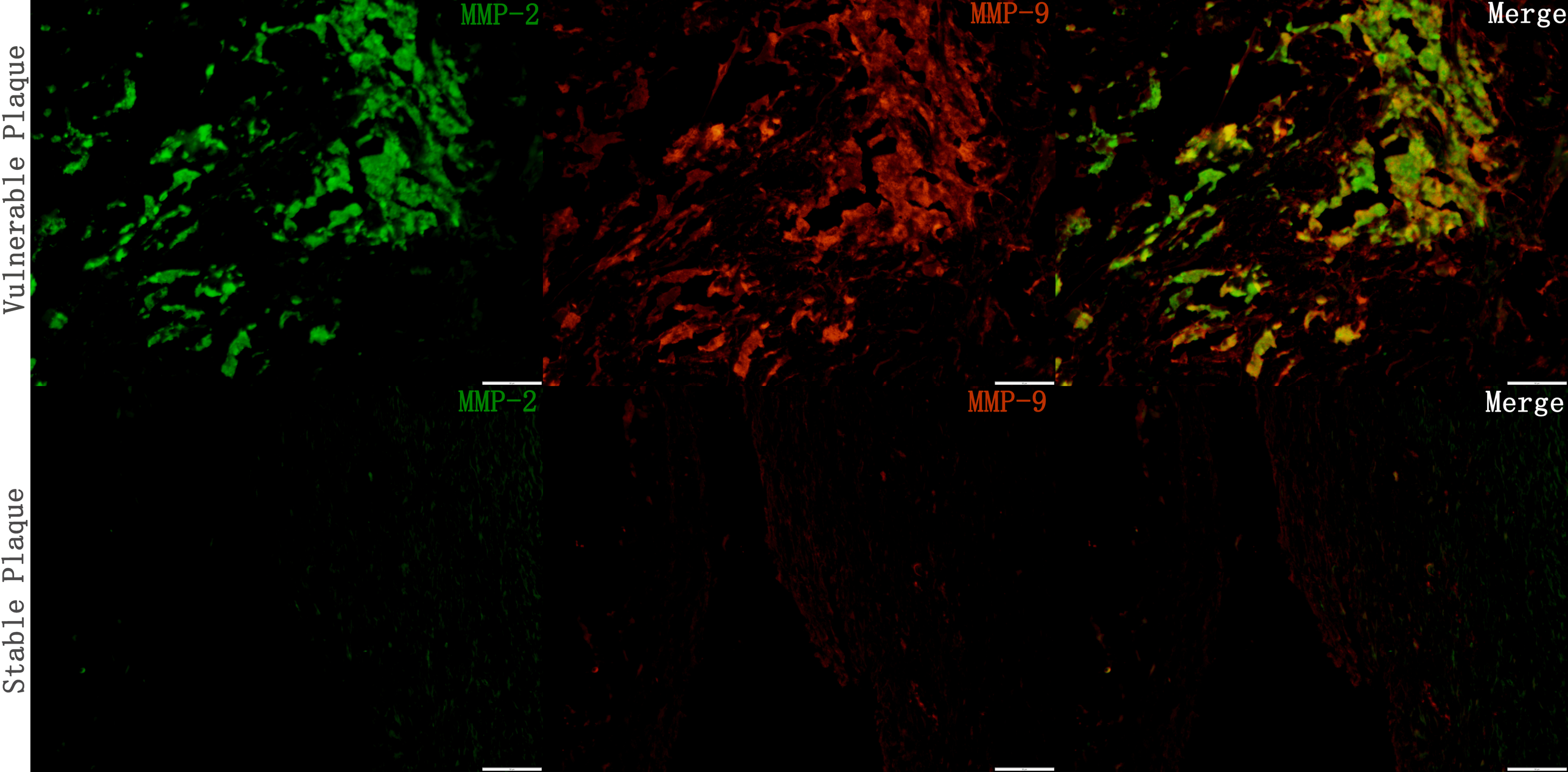

Supplement: Supplementary file 6 [file Image_3.jpeg]
